# Individual-Based Model of Microbial Life on Hydrated Rough Soil Surfaces

**DOI:** 10.1371/journal.pone.0147394

**Published:** 2016-01-25

**Authors:** Minsu Kim, Dani Or

**Affiliations:** Soil and Terrestrial Environmental Physics (STEP), Department of Environmental Systems Sciences (USYS), ETH Zürich, 8092 Zürich, Switzerland; East China Normal University, CHINA

## Abstract

Microbial life in soil is perceived as one of the most interesting ecological systems, with microbial communities exhibiting remarkable adaptability to vast dynamic environmental conditions. At the same time, it is a notoriously challenging system to understand due to its complexity including physical, chemical, and biological factors in synchrony. This study presents a spatially-resolved model of microbial dynamics on idealised rough soil surfaces represented as patches with different (roughness) properties that preserve the salient hydration physics of real surfaces. Cell level microbial interactions are considered within an individual-based formulation including dispersion and various forms of trophic dependencies (competition, mutualism). The model provides new insights into mechanisms affecting microbial community dynamics and gives rise to spontaneous formation of microbial community spatial patterns. The framework is capable of representing many interacting species and provides diversity metrics reflecting surface conditions and their evolution over time. A key feature of the model is its spatial scalability that permits representation of microbial processes from cell-level (micro-metric scales) to soil representative volumes at sub-metre scales. Several illustrative examples of microbial trophic interactions and population dynamics highlight the potential of the proposed modelling framework to quantitatively study soil microbial processes. The model is highly applicable in a wide range spanning from quantifying spatial organisation of multiple species under various hydration conditions to predicting microbial diversity residing in different soils.

## Introduction

Soil microbial activity drives some of the most globally important biogeochemical cycles (carbon, nitrogen), which are prominent in nutrient cycling in soils, purification of water, and a range of other ecosystem services [1–3]. Advances in molecular biology enabled quantification of the unparalleled diversity of microbial life making soil the most diverse and biologically active compartment of the biosphere [4–9]. The complex soil matrix supports and maintains the immense microbial diversity within physically and chemically distinctive microhabitats that are in constant state of change [10–17].

Despite the importance of soil microbial process to life on Earth, the relations between soil physical environment and microbial function remain unclear primarily due to vast heterogeneity of aqueous and chemical micro-environments, large and varying diversity of microbial life at all scales, and the complex structure of soil pores [18–20]. Field scale studies of soil microbial ecology have focused on deducing empirical relations between microbial activity and various services related to agricultural production, general ecosystem services, or climate change issues [21–26]. At the same time, progress in molecular-genetic based methods and rapid expansion in identification of microbial species were instrumental in quantifying soil diversity and population dynamics, but their application to resolving ecological questions have been limited [27–31].

A major obstacle in the utilisation of these rapidly expanding molecular genetic-based methods pertains to the dearth of quantitative frameworks for systematically interpreting microbial interactions in their natural (albeit complex) soil environmental conditions [11, 32]. An important challenge remains the disparity in the scale of processes that emanate at the level of interacting cells yet manifested at scales of ecologically relevant processes (soil profile to landscapes). The environmental conditions that affect microbial cells may vary drastically from pore to pore at micro-metric scales [33] suggesting that process representation at these scales might be important to quantifying fluxes and processes that manifest at larger scales. In addition to the spatial scale challenge, observation of microbial processes within the opaque soil matrix remains nearly impossible with present methods [34]. Recent investigations have attempted to study microbial life in soil pores using unsaturated porous ceramic surfaces [35] or micro-fluidic pore networks to observe growth of microorganisms at small scales [36], nevertheless, definitive studies of microbial function in soil pore and their small scale biogeography remain unresolved.

Modelling tools offering new insights into detailed microbial life in idealised pore spaces could bridge gaps in the present experimental limitations. Various mechanistic modelling approaches have been proposed including Individual-Based models (IBM), Lattice Boltzmann methods (LBM), and hybrid models such as IBM implemented within pore network models or on patchy surfaces [34, 37, 38]. Some of the advanced models for quantifying the spatio-temporal dynamics of microbial communities have attempted to integrate the spatial complexity of soil at the pore scale, however, these remain limited to small domains (a few millimetres), smaller than typical soil representative elementary volumes or standard sample scales.

In this study we seek to develop a scalable biophysical model for microbial function on soil rough surfaces that preserves cell-level details (micro-metric) within spatially variable roughness patches. The domain and transport processes permit representation of microbial activities at scales of up to 0.1m. The two important aspects in this modelling approach are; (1) the representation of complex soil structure with few physical measures, and (2) representing cell-level microbial response to spatial and temporal dynamics. The primary soil habitat variable in this model is the water film thickness on a rough surface patch, which regulates diffusion, dispersion and connectivity with neighbouring habitats. The scheme for generating soil rough surfaces enables control over the amount of water or water film thickness compatible with conditions in real soils (via the water characteristic curve for a soil type) in modelling microbial life (growth, interactions and dispersion) on such surfaces, we consider spatial and temporal variations in hydration conditions over small scales. In variance with standard microbial growth models in static and homogeneous conditions, the model combines these two characteristics of soil microbial life (variances in space and time) in a scalable description of surfaces as water retaining rough patches within which microbial interactions at cell to population levels are represented. The rough surface patch model (RSPM) allows upscaling of soil microbial life description from micro-metric to sub-metric domains at time-scales of seconds to months, thus supporting the representation of long-term behaviour of multiple species communities at scales of ecological relevance.

## Methods: Modelling microbial growth on hydrated rough surfaces

The necessity for quantitative modelling frameworks to advance environmental microbiology have been widely recognised [32, 39]. In the context of microbial life in soil, various modelling approaches have been proposed and used to elucidate relations between small-scale physical properties and roles of soil microbial communities in terrestrial ecosystems [37, 40, 41]. These studies provided new insights into environmental conditions that limit and promote soil bacterial life in an abstracted soil structure at very small scale (a few mm). The detailed description of pores in such models limit their upscaling to macroscopic systems that consider hydration, temperature and other gradients that shape natural populations (surface crusts, sharp fronts, etc.). To overcome this limitation while preserving cell-level description of microbial activity and their functions, we propose a rough surface patch model (RSPM) to represent natural hydrated surfaces. The modelling domain discretises the physical domain into patches that (collectively) represent soil hydration conditions at a given matric potential *ψ*_*m*_, such as effective water film thickness. A similar approach has been applied to model microbial life on pre-assigned two-dimensional roughness domain [38], however the approach was macroscopic without cell-level interactions. Soil water retention properties for each patch are described by the van Genuchten model [42] permitting consistent representation of other hydraulic functions for the patch such as unsaturated nutrient diffusion according to Millington and Quirk [43]. While such parametric representation of rough surfaces overlooks pore scale geometrical detail (sub-patch scale), it provides useful links with characteristics of soils at the sample scale while preserving spatial heterogeneity among patches, which are critical for quantifying competition and co-existence in soil.

### Representing soil rough surfaces

#### Construction of the rough surface simulation domain

The basic building block is a surface roughness patch that represents pore walls or surfaces of adjacent soil grains. Each patch contains subdomain roughness that is not explicitly represented. Instead, it is represented by self-affine and fractal properties of such water retaining surface geometries. A patch represents a multi-niche domain capable of hosting multiple species at a capacity defined by mean nutrient flux across its boundaries with neighbouring patches. The effective water film thickness and patch connectivity jointly determine local microbial cell dispersion rates (propelled by flagellated motion). The mean residence time of microbial cells in a patch varies with patch size and film thickness linked to water filled roughness (that vary with matric potential) and it can be estimated directly based on hydration conditions (For the detailed information, see S1 Text).

**Fig 1:**
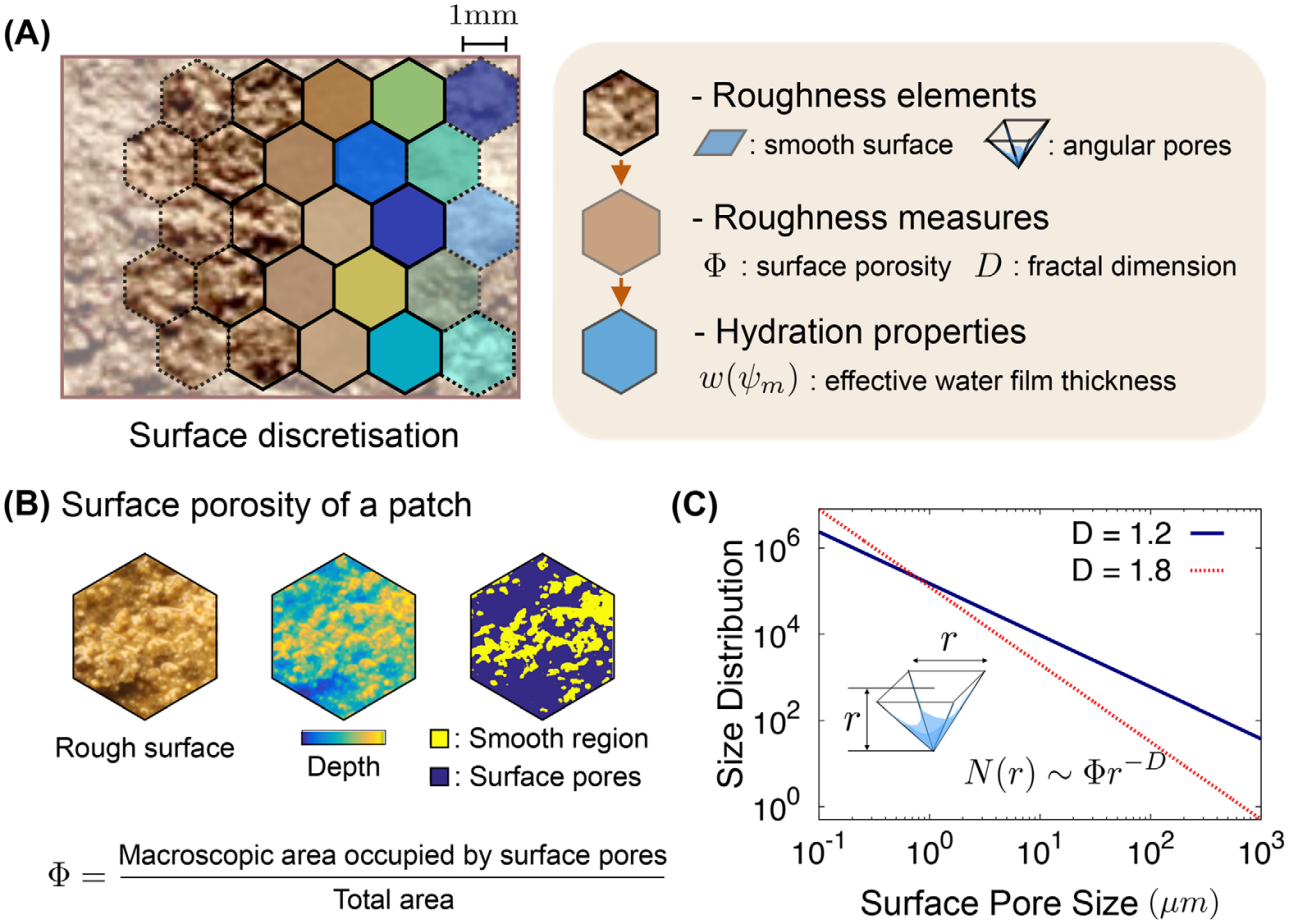
A conceptual diagram of the definition of a patch. **(A)** A rough soil surface domain is discretised with hexagonal patches representing subdomains. The brown and blue colour scale indicate the homogenisation of the roughness and hydration condition for each patch, respectively. To obtain the characteristics of patches we assume that a patch consists of roughness elements, a conceptual water-retaining pyramid-shaped pore and a smooth surface region. It allows us to calculate the amount of water held on the rough surface from the capillarity and van der Waals adsorptive forces at a certain relative humidity. Roughness of a patch is characterised with two measures, a surface porosity *Φ* and a fractal dimension *D*. The hydration condition of the patch can be represented as the effective water film thickness *w*(*ψ*_*m*_) as a function of the water matric potential *ψ*_*m*_; Eq (2). **(B)** A rough surface domain would be comprised of various size of angular surface pores on the smooth surface. Surface porosity of a patch *Φ* determines the fraction of surface pores with respect to the patch domain (smooth surface+angular pores). **(C)** The fractal dimension *D* determines the size distribution of roughness elements for a patch, *N*(*r*). It follows a power-law with a fractal dimension *D*. In the model, we assumed that the surface pore is the shape of a square pyramid with base *r* and height *r* (Larger pores indicate deeper pits on the rough surface).

The nature of roughness in each patch is characterised by a surface porosity *Φ* (roughness space for storing water) and an exponent *D* for roughness element size distribution (See a diagram for the roughness domain depicted in Fig 1).

In contrast to the surface pore network models [37, 40], the rough patch contains no geometrical details (other than average or parametric properties: See Fig 1A). However, we set a conceptual angular pore (with depth and sides r) and a smooth region as roughness elements for an explicit description of capillary force and its corner effect on surface pores. We assume that the surface pore is the shape of a square pyramid with base and depth *r* and the smooth surface is a completely smooth surface where water films are held by absorptive force only [44]. The surface porosity *Φ* represents the fraction of the surface occupied by pores (angular depressions) relative to the entire patch domain (Fig 1B). While *Φ* determines the proportion of surface pores on the domain, the exponent *D* controls the size distribution of roughness elements (the angular pyramid-shaped depressions). For spatial scalability considerations, we assume that the distribution of surface pore volumes follows a power-law distribution (Fig 1C).

$$N\left(r\right)∼r^{-D},$$ (1)

where *N*(*r*) is the surface pore size distribution with size *r*. Here, to avoid problems of divergence and to consider realistic roughness elements, upper and lower size cutoffs are introduced: *r*_min_ ≤ *r* ≤ *r*_max_. *r*_min_ is set to be 10^−7^ (m) to represent the minimum size of physical elements on the rough surface (related to the size of clay particle). *r*_max_ varies depending on the scale of the domain and the largest roughness element. We note that the power-law nature of the pore size distribution is adopted from fractal models of soil for the purpose of up-scaling patch size for a given domain. Assuming Eq (1) in its respective boundaries allows us to calculate hydration properties analytically and we thus use it for its simplicity. We call the exponent *D* as a fractal dimension for roughness measures.

Based on the fractal dimension *D* and the surface porosity *Φ*, the effective water film thickness and the degree of saturation of a patch can be calculated by averaging the distribution of each element. For example, the effective water film thickness is defined as the value of the expected total amount of water at the water matric potential *ψ*_*m*_, $\bar{V}\left(\psi_{m}\right)$, divided by the expected surface area of the patch, $\bar{A}$. Thus,

$$w_{\text{eff}}\left(\psi_{m}\right)=\frac{\bar{V}\left(\psi_{m}\right)}{\bar{A}}=\frac{\int_{r_{\text{min}}}^{r_{\text{max}}}\left[\Phi V\left(r,\psi_{m}\right)+\left(1-\Phi \right)h_{\mu }\left(\psi_{m}\right)r^{2}\right]r^{-(D+1)}dr}{\int_{r_{\text{min}}}^{r_{\text{max}}}\;r^{2}r^{-(D+1)}dr},$$ (2)

where $V(r,\psi_{m})$ is the amount of water which is held in an angular pore with size *r* and *h*_*μ*_(*ψ*_*m*_) is absorbed film thickness at *ψ*_*m*_. When Φ → 0, the surface domain contains no angular pores thus only the contribution of van der Waals adsorptive forces would be left, which would determine the film thickness on that patch. On the other hand, when Φ → 1, the surface becomes very rough without any smooth area and the distribution of depth over the domain is purely given by the fractal dimension. The amount of water and effective water film thickness are calculated under considerations on physical properties of rough surfaces only; however, the model can be modified by considering chemical or biological agents that affect to the surface property. For instance, surfactant production by micro-organisms can alter the water-retention curve by lowering surface tension and increasing contact angle on the surface [45, 46]. In this work, we did not consider these effects for sake of simplicity and calculated hydration property of surfaces with the surface tension of water, 72mN.m^-1^, and the contact angle as 0° (For the detailed calculations, see S1 Text).

Spatial variations and heterogeneity of the simulation domain are represented by assigning roughness measures to each patch drawn from a distribution of parameter values that preserve mean soil behaviour. As we have shown, a set of parameters {Φ, *D*} fully determines hydration properties of each patch in the model.

For the fractal dimension in the present work, we made an assumption that *D* is constant for the entire domain. Most of fractal models on soil structure distinguish fractal dimensions of mass *D*_*m*_, pore volume *D*_*p*_, and surface pore *D*_*s*_ in terms of size distributions [47, 48]. To simplify modelling the soil structure in the present work, we assume that *D* = *D*_*s*_ = *D*_*m*_ − 1 = *D*_*p*_ − 1 as a constant for the entire domain and analogously interpret that *D* determines different types or textures of soils [49–52]. For bulk soils, most of studies agree that sandy soils exhibit lower fractal dimension about *D*_*p*_ ≈ 2.4 and higher clay contents increases *D*_*p*_ close to 3 [49]. Furthermore, some studies have provided measured data on fractal dimensions of soil surfaces to describe shape and form of natural objects as habitats of soil organisms such as micro-arthropod [53, 54], earthworms [55], protozoa and bacterial species [56]. Especially in the work of [53], the fractal dimension is measured from two-dimensional soil sections (in mm scales) by using image analysis technique. The size of patches (size of surface pores in our model) follows a power-law distribution with *D* ≈ 1.4. The work of [55] also measured the surface pore size distribution of the soil sections in cm scales and showed that *D* ranged between 1.32 and 1.70. The fractal dimension *D* for surface pore size distribution in RSPM is also in the range of 1 < *D* < 2 and it can be connected with the surface fractal dimension describing the surface roughness [57].

**Fig 2:**
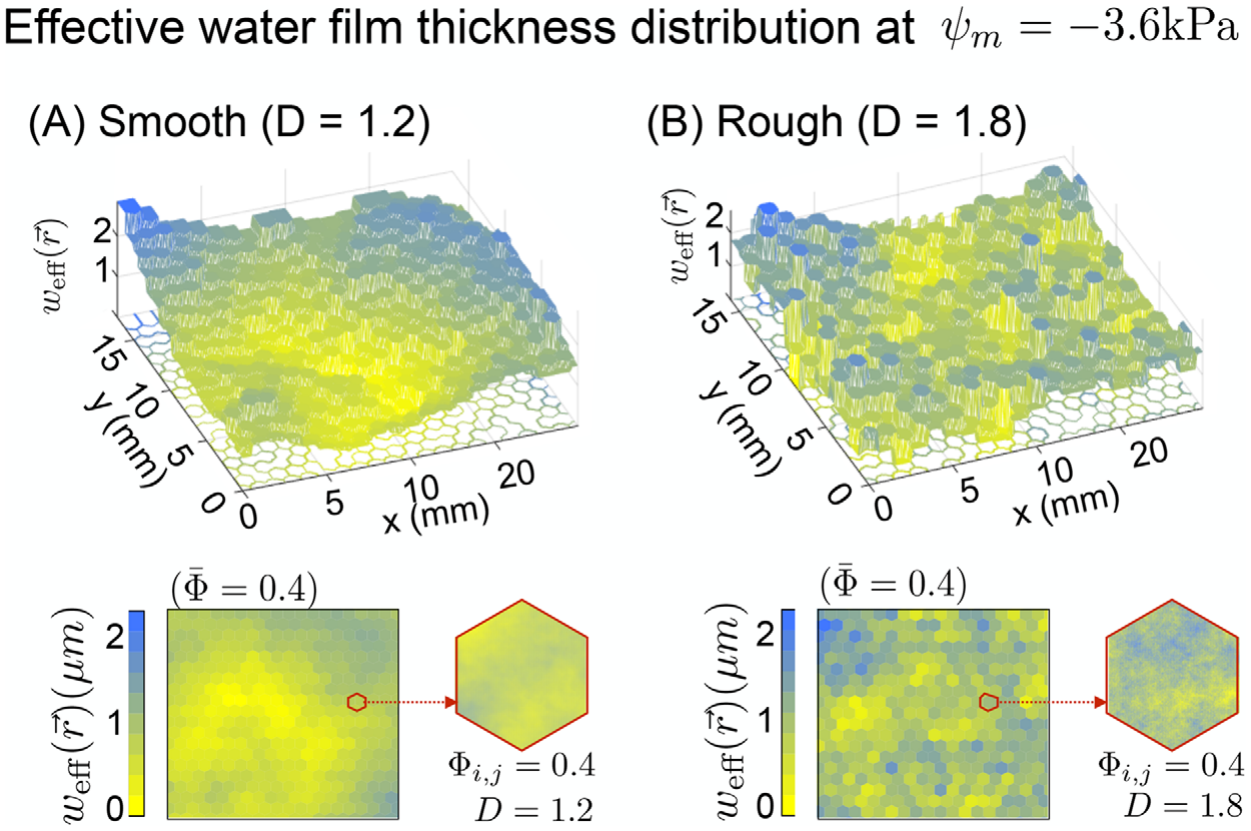
A comparison of effective water film thickness distribution $w_{\text{eff}}(\vec{r})$ between smooth and rough surface domain. Typical examples of rough surface domain are given as the effective water film thickness distribution at *ψ*_*m*_ = -3.6kPa. **(A)** a smooth surface domain (*D* = 1.2) and **(B)** a rough surface domain (*D* = 1.8) are provided for a comparison. To illustrate the role of the fractal dimension *D* for generating self-affine characteristics, the mean surface porosity of the domain is fixed as a constant for both cases ($\bar{\Phi }=0.4$). A patch in the domain is assumed to be homogeneous inside for its roughness and hydration condition (effective water film thickness). However, to incorporate its roughness into the connectivity and tortuosity of the hydrological pathways in the patch, the global percolation probability of the domain and the local surface porosity Φ_*i*,*j*_ are considered to determine the residence time of microorganisms at the patch.

For the rough surface properties, the fractal dimension controls the specific surface area of the domain and the size distribution of surface pores. Thus, only one parameter, the surface porosity Φ, which is the proportion of area occupied by angular pores on the macroscopic surface area, is used to assign the heterogeneity of the roughness domain. However, any random spatial distribution of the local surface porosity cannot guarantee the persistence of up-scalability at the domain scale. To match the system domain with the soil texture and surface roughness maintaining simple scalability, we assumed the surface porosity distribution follows the self-affine structure [57, 58]. Eq (2) shows that the effective water film thickness of a patch is linearly dependent on surface porosity Φ. It implies that the length scale that we concern for substrate diffusion and microbial dispersion can be solely determined by Φ when *D* is constant. From the linear relation between the effective water film thickness and the surface porosity, we applied the definition of self-affinity relating the horizontal displacement Δ*r* (distance) and the vertical displacement Δ*z* (depth) [59, 60],

$$\Delta z∼\Delta w_{\text{eff}}∼\Delta \Phi ∼{\left(\Delta r\right)}^{H}\equiv {\left(\Delta r\right)}^{3-Dp}={\left(\Delta r\right)}^{2-D},$$ (3)

where *H* ∈ [0, 1] is the Hurst’s exponent (*H* = 2 − *D*). The distribution of the surface porosity for the domain is obtained by implemeting fractional Brownian surfaces [61, 62] that preserve the relation, Eq (3), with the mean value over the domain $\bar{\Phi }$ within a bounded region, $\Phi \left(\vec{r}\right)\in \left[0,1\right]$. Typical examples of the surface domain for different roughness are given in Fig 2. In the figure, the roughness of the domain is depicted as the effective water film thickness distribution. As a patch model, each patch is assumed to be homogeneous inside and its roughness and hydration properties are averaged following the probability distribution. In terms of connectivity, we assumed the patch is a small replica of the entire domain (statistically self-similar to achieve the scalability) and the residence time of the microorganisms and the degree of interactions are determined by the global percolation probability of aqueous phase and the local surface porosity (This will be discussed in the section: Hydration and fragmentation of aqueous habitats.). To sum up, the spatial heterogeneity of the local surface porosity with the self-affinity allows us to obtain distributions of available water (locally at the patch scale) in terms of saturation degree and effective water film thickness simultaneously with the representative roughness measures. This determines the local carrying capacity of microorganisms under a certain hydration condition and the nutrient flux from adjacent patches.

#### Diffusion process on rough surface domain

Nutrient diffusion variations and limitations at the micro-scale is an important mechanism for diverse microbial activity at small scales in soil. The nutrient flux to a physical niche determines the carrying capacity and various trophic interactions among species. The model links between micro-hydrology nutrient diffusion and dispersal of microorganisms on rough surfaces. In the model, transport properties on the rough surface are described explicitly and local conditions modify the development of microbial individuals. This modification at individual development would have a significant impact on community activity and this process regulates the spatial distribution of the microorganisms and the physical and chemical properties of the habitat.

Since we average microscopic details of the surface pore distribution and assume that hydration conditions are represented with effective water film thickness, the diffusion process is also described in terms of this parameter. The flux into a patch is estimated considering the cross section between adjacent patches based on their effective film thickness. In case that the effective film thickness among two neighbouring patches are different, we choose the minimum value of between them to guarantee that the joint cross sectional area is dictated by the thinner film. We apply Fick’s law to calculate local substrate concentration, $C(\vec{r},t)$, considering the reaction diffusion equation,

$$\frac{\partial C(\vec{r},t)}{\partial t}=\nabla \cdot \left(D\left(\vec{r}\right)\nabla C\left(\vec{r},t\right)\right)-\frac{1}{V_{w}\left(\vec{r}\right)}f\left(b\left(\vec{r}\right),C\left(\vec{r},t\right)\right),$$ (4)

where $D(\vec{r})$ is the apparent diffusion coefficient defined from the effective film thickness distribution of adjacent patches. Detailed calculation is presentred in S1 Text. In Eq (4), *f*(*b*, *C*) is the total mass consumption by microorganisms with the total biomass *b* at the patch with the position $\vec{r}$. $V_{w}\left(\vec{r}\right)$ is the volume of water in the patch (area of the patch × the effective film thickness), thus the second term on r.h.s. indicates the change of concentration as a reaction term.

### Microbial growth on rough surfaces

The present model employs Individual-Based Modelling (IBM) to describe dynamics of microbial activity on heterogeneous rough surface [63, 64]. The IBM is capable of capturing interactions among cells competing for nutrients, or other forms of trophic dependencies such as mutualistic interactions between species (at the cell level). IBM represents cell-response to the physicochemical micro-environments with high spatial and temporal variations [65]. Although implementation of IBM requires considerable parameterisation that distinguishes physiological traits of various species (often derived from experimental results), the trophic preferences and interactions among species give rise to emergence of spatial patterns and ecological functionality in complex spatial domains is a distinct advantage [66–68].

Life on the patchy rough surface merges IBM with the generalised physiological characteristics of bacterial cells, such as substrate uptake rates, metabolism, maintenance, reproduction, chemotactic motion, and death [69]. The growth of an individual cell is determined from the local concentration. Lack of nutrients for a certain period exhaust cell reserves and lead to cell death following rules based on previous studies [69]. The model is spatially explicit and includes microbial motility that is regulated by balancing the capillary force on surface and the chemotactic motion of the microbial cell.

Considering the substrate consumption rates by microbial cells, the reaction diffusion Eq (4) can be rewritten explicitly as

$$\frac{\partial C(\vec{r},t)}{\partial t}=\nabla \cdot \left(D\left(\vec{r}\right)\nabla C\left(\vec{r},t\right)\right)-\frac{1}{V_{w}\left(\vec{r}\right)}\sum_{i=1}^{N(\vec{r},t)}f_{p}\left(\vec{r}\right)\frac{\mu_{i}\left(\vec{r},t\right)}{Y^{i}{}_{\text{max}}}b_{i}\left(\vec{r},t\right),$$ (5)

where $N(\vec{r},t)$ is the number of individual cells in the patch at $\vec{r}$, $Y^{i}{}_{\text{max}}$ is the maximum growth yield with respect to the substrate, and $b_{i}\left(\vec{r},t\right)$ is the biomass of cell *i* at time *t*. This implies that the reaction term is the total consumption of the nutrient by every individual at the patch. *μ*_*i*_ is the specific growth rate of the microbial cell *i* defined with Monod growth function [70, 71]

$$\mu_{i}\left(\vec{r},t\right)=\frac{\mu^{i}{}_{\text{max}}C\left(\vec{r},t\right)}{K_{s,i}+C\left(\vec{r},t\right)},$$ (6)

where $\mu^{i}{}_{\text{max}}$, *K*_*s*,*i*_ are the maximum growth rate and the half-saturation constant of the cell *i*, respectively. Here, although the Monod equation is generally used for population growth on batch culture, it is known that the single cell growth also follows the same Eq [36]. Monod growth can be extended in the model for multiple nutrients [72]. When several nutrients are limiting the growth rate of the cell, the change of biomass *b*_*i*_ can be written as

$$\begin{array}{ccc} {\tilde{\mu }}^{i}b_{i}=\frac{db_{i}}{dt} & = & \left(\mu^{i}{}_{\text{max}}\;\min\left[\left\{\frac{C_{1}}{K_{s,i}^{1}+C_{1}},\frac{C_{2}}{K_{s,i}^{2}+C_{2}},\cdots \right\}\right]-m_{i}\right)b_{i},\\ {\tilde{\mu }}^{i} & = & \mu^{i}{}_{\text{max}}\;\min\left[\left\{\frac{C_{1}}{K_{s,i}^{1}+C_{1}},\frac{C_{2}}{K_{s,i}^{2}+C_{2}},\cdots \right\}\right]-m_{i}=\mu_{i}-m_{i}, \end{array}$$ (7)

where *C*_*j*_ indicates the j-th limiting nutrient, $K_{s,i}^{j}$ is the half saturation constant with respect to the nutrient *j*, and *m*_*i*_ is the maintenance rate of the cell *i*. Here, we assume that the maintenance rate of cell *i* is proportional to its maximum growth rate, *m*_*i*_ ≡ *α*_*m*_ *μ*^*i*^_max_.

A similar reaction-diffusion formulation has been used in previous studies of microbial growth on roughness network models, and pore network models [37, 38, 41]. Unlike previous studies, the patch definition of a spatial element is not a single niche and the model is scalable (capable of representing complex gradients over sub-metric scales), thus, the nutrient consumption within a patch also depends on roughness elements distribution and connections to the domain boundaries. Although the nutrient concentration or hydration condition is assumed to be constant for all individuals located at the same patch, the accessibility to the substrate and the degree of interaction among cells is not uniform. Considering this, we introduce in Eq (5) a factor $f_{p}\left(\vec{r}\right)$ as the nutrients sharing factor. This factor considers the connectivity of the surface within a patch that cannot be expressed explicitly due to the spatial averaging representation (essential for effective upscaling). We assume that the nutrient sharing factor combines the deterministic microbial consumption rates with a local stochastic component via

$$f_{p}\left(\vec{r}\right)=\xi \left(\psi_{m},\vec{r}\right)+\chi_{p}\left(1-\xi \left(\psi_{m},\vec{r}\right)\right),$$ (8)

where *χ*_*p*_ is a random number drawn from a uniform distribution, U[0,1]. To consider different nutritional environments within a patch without burdening the computations with geometrical detail, we employ stochastic nutrient sharing assigned based on the level of local connectivity at the patch scale, $\xi \left(\psi_{m},\vec{r}\right)\in \left[0,1\right]$. The estimation of local connectivity within a patch will be discussed in the next section. For $\xi (\psi_{m},\vec{r})\to 1$, that is, when the local domain (a patch) is fully connected and microorganisms can access the entire region by flagellated motion with no restrictions of surface and other abiotic structures, the nutrient sharing factor becomes unity and consumption rates by each organism are determined solely by their respective growth function, Eq (6). Under such conditions (static and fully connected) with many microbial species, the species with the highest $\mu^{i}{}_{\text{max}}$ and the lowest *K*_*s*,*i*_ will dominate the patch at long time scale (the patch represents a single niche) [73–77]. On the other hand, as $\xi (\psi_{m},\vec{r})\to 0$, a patch becomes highly fragmented or all the microorganisms become sessile and the consumption of nutrients for each species is diffusion based and stochastic. This stochasticity can be interpreted as unique spatial locations within a patch and reflect inherent irregularity of soil niches (even at the micro-scale) in terms of diffusion and other factors to locations where microorganisms are attached.

### Hydration and fragmentation of aqueous habitats

Surface hydration conditions play an important role in all of the microbial life functions ranging from the control of diffusion rates, habitat connectivity to cell dispersion rates and ranges. Field scale models often treat microbial dispersion as passive convection or diffusion of passive substances [78–83]. However, at the pore scale, microorganisms are not passive and actively seek nutrients and enhance their survivability by chemotaxis [84–87]. Microbial cells move on surfaces by various mechanisms including swimming and swarming by flagella, twitching, gliding, sliding, and darting [88]. Generally, surface motility is enhanced under wet conditions, especially for microbes propelled via flagellated motility where swimming speeds have shown to be sensitive to water film thickness [37, 89]. While cell swimming speed depends on physical properties (film thickness, cell sizes) [41], chemotaxis determines the direction of swimming by chemical responses to nutrient concentrations or signals from other cells. The model applied the locomotion at a single-cell level based on hydration conditions and chemotaxis.

#### Chemotactic microbial locomotion on rough surfaces

We employed the receptor model [90] to derive the specific growth rate as the chemotactic potential. This approach allows consideration of chemotactic motion in response to gradients of multiple nutrients collapsed into a single scalar potential (motion towards the direction that produces the highest specific growth rate). (For more detailed explanations, see S1 Text). A biased-random walk approach is used with the probability to cross to adjacent patches defined by the composite chemotactic field derived from local specific growth rate

$$p_{i}\left(t\right)=\frac{w_{i}e^{\alpha \left(w_{i}\right)\vec{\nabla }\mu \left(t\right)\cdot {\hat{e}}_{i}}}{\sum_{j=1}^{7}w_{j}e^{\alpha \left(w_{j}\right)\vec{\nabla }\mu \left(t\right)\cdot {\hat{e}}_{j}}},$$ (9)

when *v*(*ψ*_*m*_)≠0 and where *α* is the factor for the chemotactic motion, given by

$$\alpha \equiv \frac{\chi_{0}}{2\mu_{\text{max}}v\left(\psi_{m}\right)},$$ (10)

which balances the chemotactic sensitivity *χ*_0_ and the swimming speed of a microbial cell *v*(*ψ*_*m*_). In Eq (9), *w*_*i*_ denotes the effective film thickness of the nearest patch in the direction *i* ∈ {1, ⋯, 7} and *i* = 7 denotes the current patch where the cell locates. This implies that the motion in the patch depends on the local gradient of the chemotactic field and concurrently on the nutrient flux from different directions.

The swimming speed *v*(*ψ*_*m*_) is determined as a function of effective water film thickness *w*(*ψ*_*m*_) and it includes mechanical interaction between the surface and the microbial cell following the previous model [37]:

$$v\left(w\left(\psi_{m}\right)\right)=v_{0}\frac{\left(F_{M}-F_{\lambda }\left(w\left(\psi_{m}\right)\right)-F_{c}\left(w\left(\psi_{m}\right)\right)\right)}{F_{M}},$$ (11)

where *v*_0_ is the maximum swimming speed of a cell in bulk water. *F*_*M*_, *F*_*λ*_, *F*_*c*_ are flagellated propulsion, cell-surface hydrodynamic interaction, and capillary pinning force in the aqueous film, respectively. *F*_*λ*_ and *F*_*c*_ are the function of *w*(*ψ*_*m*_) that reflects the hydration condition and roughness element distribution. When *F*_*M*_ − *F*_*λ*_(*w*) − *F*_*c*_(*w*)<0, the capillary force becomes dominant and swimming velocity ceases (i.e. the microbial cell becomes sessile). Application of chemotactically-driven biased random walk for microbial cell displacement determines the expected travelling length and the residence time within a patch. When the expected travelling length becomes longer than *l*_*p*_/*ξ*(*ψ*_*m*_), the bacterial cell moves to the other patch based on cumulated location. We assume that the inverse of *ξ*(*ψ*_*m*_), the local connectivity of the patch, to be the tortuosity of the patch, $\tau \left(\psi_{m}\right)\equiv \frac{1}{\xi (\psi_{m})}$.

The minimum residence time of a bacterial cell in a patch $T_{r}\left(\vec{r},\psi_{m}\right)$ is defined to represent the contribution of surface roughness to microbial dispersion as a physical property regardless of the chemical conditions such as substrate concentration that controls the chemotactic behaviour. We define the averaged minimum residence time ${\bar{T}}_{r}$ of the domain at *ψ*_*m*_ as following,

$${\bar{T}}_{r}\left(\psi_{m}\right)=\frac{1}{\int_{\Omega }d\Omega }\int_{\Omega }\frac{l_{p}}{v\left(\vec{r},\psi_{m}\right)\xi \left(\vec{r},\psi_{m}\right)}d\Omega$$ (12)

where *l*_*p*_ is the size of a patch and *Ω* is the system domain. This is the spatial average of the time to travel the hydrological pathways with the speed $v(\vec{r},\psi_{m})$.

#### Fragmentation of aquatic habitats on surfaces

In our model, the notion of aqueous phase connectivity on the rough surface considers two aspects; nutrient diffusion via the liquid phase and microbial dispersion rates and ranges. The structural effect of hydrological connectivities for nutrients is already averaged in terms of effective film thickness [91]. The connectivity for microbial dispersion is treated differently from nutrient diffusion especially as microbial cell sizes become comparable to surface film thickness under mild matric potential values (micro-meteric at a few kilopascal) that limit dispersion by surface capillarity long before nutrient (molecular scale) diffusion becomes limiting.

We thus define “aqueous habitats” as aqueous surface regions bounded by thin films (too thin to support flagellated motion, but sufficient to support nutrient diffusion) or physical ridges preventing accessibility of microbes external to the connected aqueous cluster—which may consists of several patches. Microbial motility within a habitat may be supported or suppressed by local (patch scale) water film thickness. In the proposed RSPM, the hydration status of each patch with respect to motility is defined as either motile or sessile based on two criteria: (1) microbial motility is enabled by a sufficiently thick aqueous film. In other words, a “motile” patch is defined on the basis that microbial swimming velocity, Eq (11), is nonzero. (2) connectivity within the patch should be high enough so microbial cells can percolate though the patch. This can be calculated following the expected occupation probability of accessible surface pores by flagellated motion. This explains the effects of physical landscape that affects travelling pathways of microbial cells and determine the minimum residence time within a patch (For the detailed information, see S1 Text). Hence, a patch is classified as “motile” when flagellated motion is supported by water film and the occupation probability of accessible pore regions is higher than percolation threshold at the patch so the connectivity within the patch is not zero (i.e. $v(w\left(\vec{r},\psi_{m}\right))>0$ and $T_{r}\left(\vec{r},\psi_{m}\right)$ is finite.). When these two criterion were not satisfied, the patch would be a “sessile” patch. Aqueous habitats in RSPM represent the collection of motile patches that allows microbial migration between patches. The distribution of roughness measures and their hydration properties (ability to retain water), and shapes the size and connectivity of aquatic habitats of microorganisms at domain scale.

However, the occupation probability of accessible pore regions cannot be fulfilled to describe the local connectivity within a patch since the structural information (such as the arrangement of surface pores) would be lost from the spatial averaging method with probability density function of element size ($N\left(r\right)∼\Phi r^{-D}$). To compensate this, we retrieve the spatial information of the patch by assuming the scale invariant property on local connectivity. To clarify, there are two distinctive concepts of connectedness; local connectivity (within a patch) and global connectivity of aqueous habitats (among patches). We defined the local connectivity $\xi (\psi_{m},\vec{r})$ of the patch at $\vec{r}$ by using the occupation probability of accessible pore regions of the patch $p(\psi_{m},\vec{r})$ and the global percolation probability of aqueous habitats *P*(*ψ*_*m*_) that is the proportion of the largest cluster region to the entire domain.

$$\xi \left(\psi_{m},\vec{r}\right)=\left\{\begin{array}{cc} P(\psi_{m}) & \text{if}\;p\left(\psi_{m},\vec{r}\right)>p_{c}\left(\vec{r}\right)\\ p\left(\psi_{m},\vec{r}\right)P\left(\psi_{m}\right) & \text{elsewhere} \end{array}\right.,$$

where $p_{c}\left(\vec{r}\right)$ is the percolation threshold of the patch on self-affine surface. Previous studies have shown that the percolation threshold *p*_*c*_ on a self-affine surface is dependent on the Hurst’s exponent *H* (roughness parameter) and *p*_*c*_ is a stochastic variable with a mean value (ensemble averaged) 〈*p*_*c*_〉(*H*) and a variance *σ*(*H*) regardless of system sizes [92–95]. The mean value of 〈*p*_*c*_〉(*H*) monotonically decreases with *H* such that 〈*p*_*c*_〉(*H* = 0) = 0.5 and 〈*p*_*c*_〉(*H* = 1) = 0.386 [96]. For example, a self-affine domain with *H* = 0.2 (*D* = 1.8) would have a percolation threshold value around 〈*p*_*c*_〉(0.2) ∼ 0.46 ± 0.08 regardless of system size [94]. Thus, we draw a certain local percolation threshold value $p_{c}\left(\vec{r}\right)$ for each patch from a normal distribution with mean 〈*p*_*c*_〉(*H*) and a variance *σ*(*H*). *P*(*ψ*_*m*_) the global connectivity (i.e. the probability that a patch belongs to the percolating cluster of aqueous habitats) relates the local surface property and the global surface property in terms of roughness. (See detailed description in S1 Text).

## Results

### Effective water film thickness of rough surfaces

We first consider the physical properties of an individual patch. We examined relationships between the surface porosity Φ on the effective film thickness considering that surface porosity is used to generate modelling domain heterogeneity (spatial distribution of patches with different properties). Model predictions were compared with measurements of Tokunaga and Wan [97] for film thickness measurements of a rough rock surface. In that study, the averaged film thickness was calculated by taking the difference between smooth- and rough-surface blocks and dividing by macroscopic surface area. The approach is used in our model definition of the effective film thickness. The linear averaging over the surface considers the contributions of very thick films and very thin films based on their surface pore distribution and depression of the surface.

**Fig 3:**
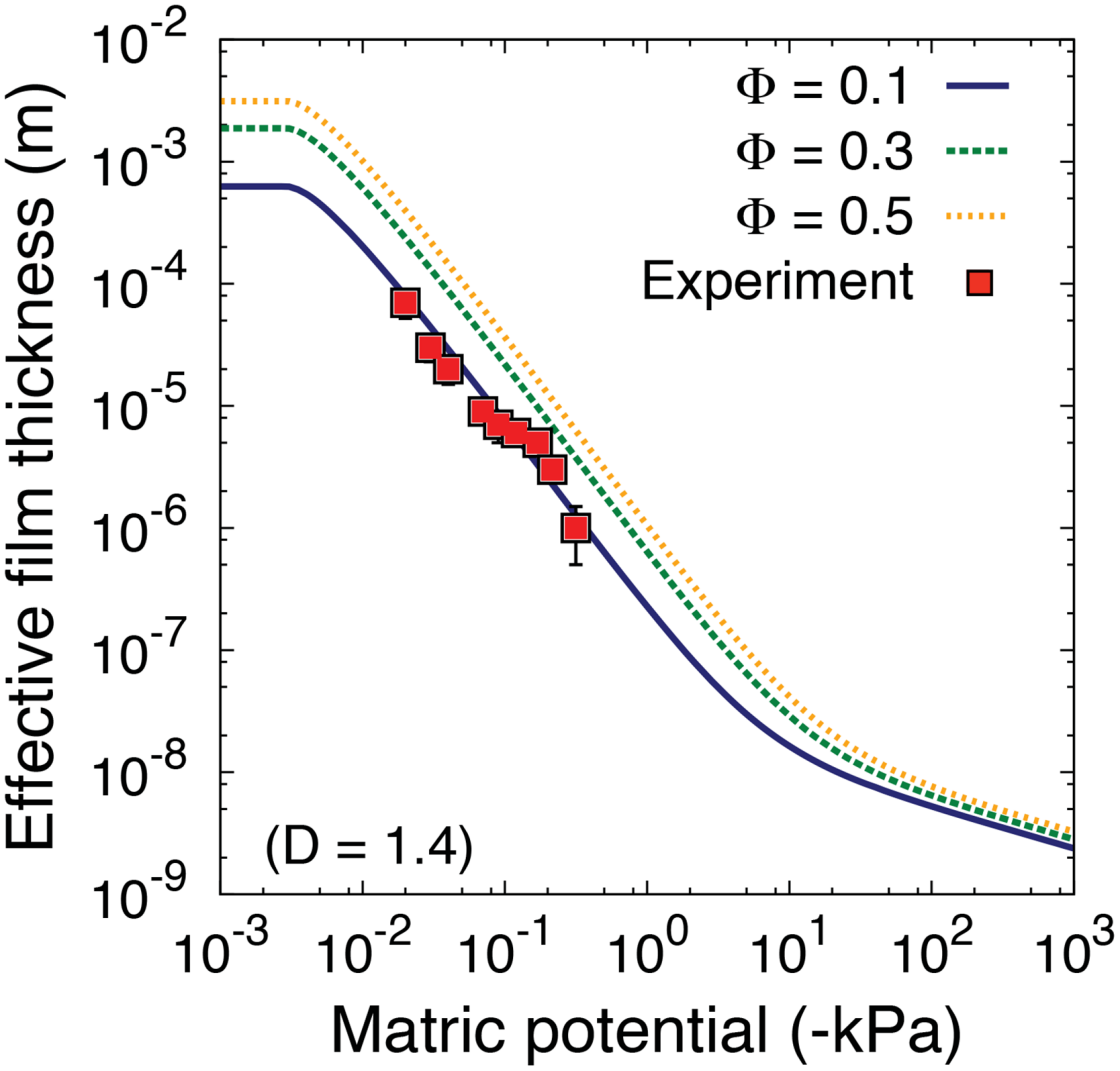
The effective water film thickness of the rough surface domain as a single patch for different hydration conditions (expressed by the matric potential of the aqueous phase). The surface porosity scales effective water film thickness when the fractal dimension is constant *D* = 1.4 (*D*_*p*_ = 2.4). When the surface porosity (Φ = 0.1) is low the model agrees with the experimental data of [97]. Here, we set the largest roughness element size *r*_max_ = 50 mm as a possible representation of the surface depression of the sample rock used in the experiment [97].

In Fig 3, a comparison with the model and the experimental data is given. The figure shows that the experimental data (Bishop Tuff, porous fractured rock with the sample size ≈50mm) of effective water film thickness from [97] agree with the model when the surface has a relatively low surface porosity (Φ = 0.1) when *D* = 1.4. Additionally, one can observe that the effective water film thickness at saturation reaches a certain value (when *ψ*_m_ → 0). The value is statistically averaged with water-filled pores and absorbed water film over the domain. As the surface becomes drier, surface pores gradually become desaturated and only absorbed film (*w*_eff_ → a few nanometres, a few number of water molecular layers) remains on the surface and held by van der Waals forces, *ψ*_*m*_ ≪ −10^3^kPa [98].

### Microbial cell mean flagellated propulsion speed

**Fig 4:**
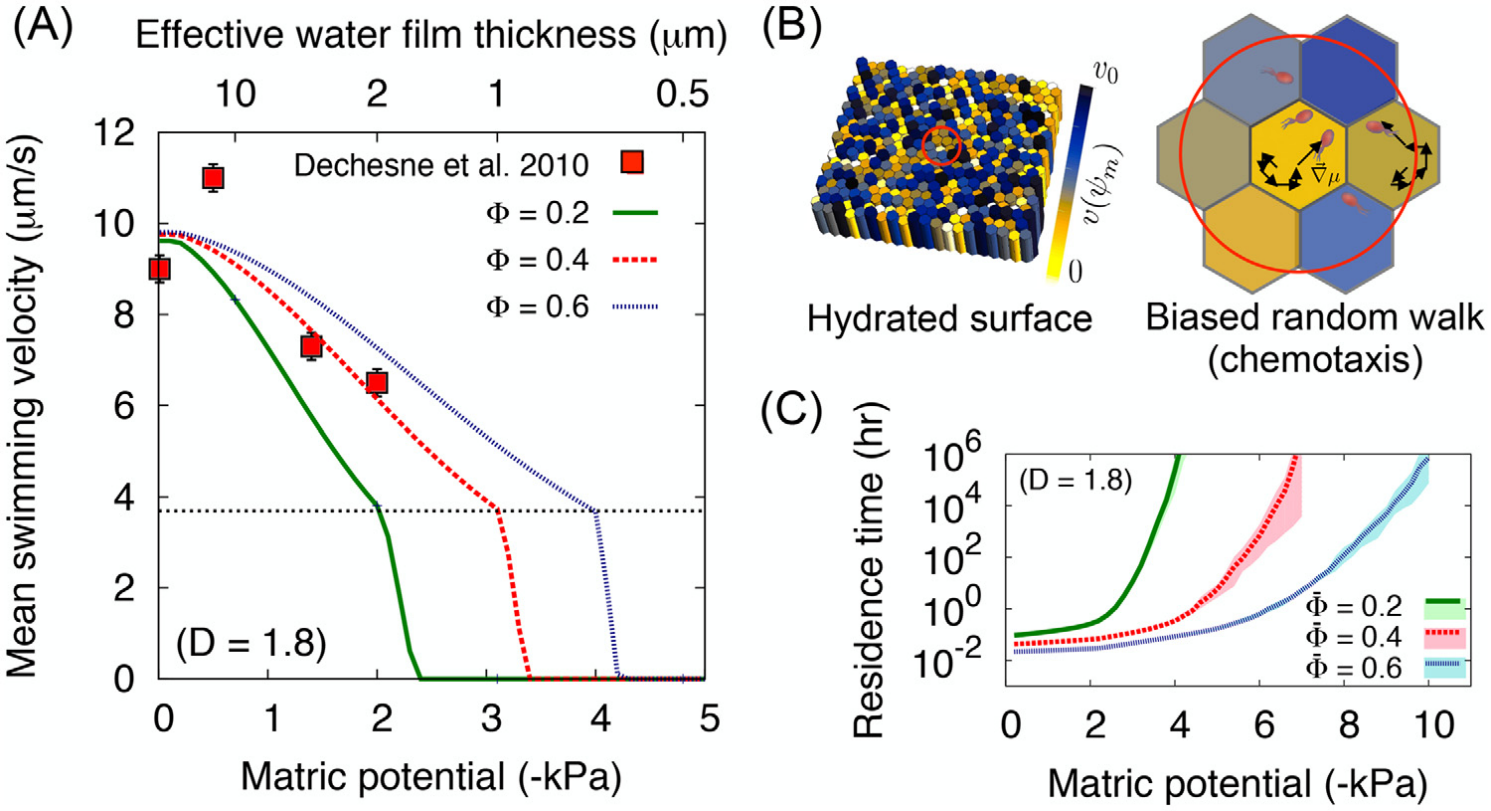
Microbial locomotion in rough surface patch model. (A) The mean flagellated swimming velocity on the surface with different surface porosities for different hydration conditions expressed by matric potential (bottom axis) and effective water film thickness when Φ = 0.4 (top axis). For comparison, we fixed the fractal dimension *D* = 1.8 and varied the surface porosity from 0.2 to 0.6. Measured values (red squares) from the work of [89], the mean microbial swimming velocity on the porous ceramic plate, show good agreement with the model when the surface porosity is about 0.4. Black dotted horizontal line indicates the onset of capillary force. The swimming speed at the bulk water is given as *v*_0_ = 14*μ*m/s [85]. The roughness effect and the surface porosity reduce the mean swimming velocity to about 10*μ*m/s even at the very wet case. (B) Heterogeneity of roughness patches on the domain can be mapped to the swimming velocity field for micro-organisms. Yellow-blue scale indicates the mean swimming speed. In a patch, the microbial locomotion follows the biased random walk following the probability to cross to adjacent patches, Eq (9). (C) The averaged minimum residence time at a patch ${\bar{T}}_{r}$ (assuming patch size *l*_*p*_ = 500*μ*m) varies for different roughness measures, Eq (12). For a surface with constant fractal dimension, the averaged minimum residence time at a patch is higher when the surface porosity $\bar{\Phi }$ is lower. The shaded area indicate the lower and upper values from 5 sample domains with the same mean roughness measures $\left\{\bar{\Phi },D\right\}$.

The effective film thickness determines the microbial swimming speed on the surface. At local patch scale, the roughness defines how a patch affects the mean swimming velocity, thus determines the mean residence time at the given patch. A typical result of the mean swimming speed for different roughness measures is given in Fig 4A. The figure shows the effect of surface porosity when *D* is constant and the capillary pinning force for flagella movement; the reduction of maximum swimming speed. Unlike the previous studies on the roughness network model [37, 40], assigning channel angles or height is not necessary since the effective water film thickness already averages the heterogeneous surface domain by using the probability distribution function of the surface pore sizes. The result shows that surface capillary force plays the most dominant role for microbial motility. The experimental data on a porous ceramic plate of [89] agree well with the model surface of *D* = 1.8 and Φ = 0.4. Here, we fixed *r*_max_ = 10^−3^ m considering the the size of ceramic surface used by [89] and the absence of large roughness elements unlike natural rocks like Bishop Tuff used in the previous section [97].

Considering that the mean swimming speed in the experiment of [89] is obtained by averaging swimming speeds of microbial cells during phases of significant motility over the entire domain, it is reasonable to expect that spatial heterogeneity was also averaged. This implies that the spatial average, ensemble average for simulations, and statistical average of individual motion for many cells (at population level) would behave identically.

So far, we have shown properties of an individual patch as an element of the physical domain. The model agrees with experimental data of effective water film thickness and the mean motile cells swimming velocity. For this physical property analysis, the size of patch does not play any role since the effective water film thickness and mean swimming velocity are intensive quantities, in other words, these values are independent of system size because a probability distribution (described with {Φ, *D*}) is used for spatial averaging. However, to simulate dynamics of microbial populations, assigning the size of patch is necessary. In the model, we set the size of a hexagonal patch as 500*μ*m, both for computational purposes and for a comparison with the experimental data. The physical domain of the model is comprised of 100 × 100 hexagonal patches and thus the entire domain size is about 5cm. In addition, we include surface spatial heterogeneity by applying different roughness parameters for each patch. For simulations, the fractal dimension is fixed, *D* = 1.8 and the heterogeneity of the surface porosity are assigned to represent a self-affine roughness when *H* = 0.2 with the mean surface porosity of all patches, $\bar{\Phi }=0.4$ (See Fig 2A for an example domain mapped to the effective film thickness distribution). The surface porosity distribution determines the swimming velocity field that controls the microbial dispersion rate by balancing capillarity and the chemotaxis (described as a biased-random walk) (See Fig 4B).

The effect of surface porosity on the minimum residence time of microbial cells with a patch of size *l*_*p*_ = 500*μ*m for different hydration conditions (matric potential values) is depicted in Fig 4C. The results show that lower surface porosities results in an increased residence times. The residence time diverges at a certain *ψ*_*m*_ due to the onset of capillary pinning forces and the fragmentation of the aqueous habitats. However, ${\bar{T}}_{r}$ represents only the minimum residence time of microbial cells. In the model, the actual residence time varies depending on the substrate diffusion and the nutrient concentration of each patch. The actual dispersal of microbial cells and their distributions are highly dependent on the chemotaxis as we inllustrate in the next section.

### Microbial dispersion rates

**Fig 5:**
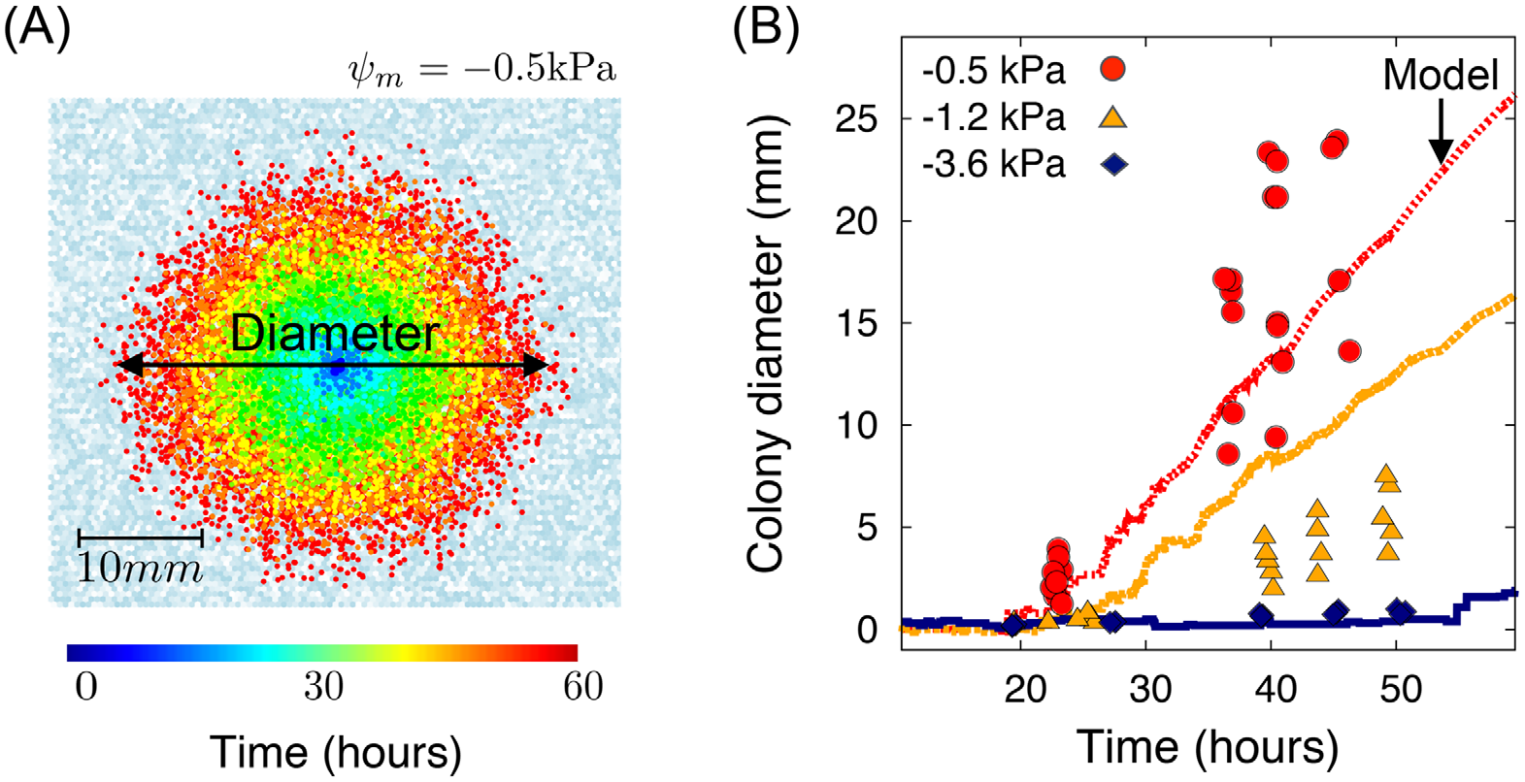
Microbial dispersion on rough surfaces. (A) Simulated colony expansion of motile bacterial cells grown in a surface at *ψ*_*m*_ = −0.5kPa. The white-greyblue colour scale indicate the effective water film thickness distribution (blue = motile, white = sessile), Here, we did not use self-affine domain for the local surface porosity distribution to reflect the experimental setting of [35].). The initial nutrient concentration was given $C\left(\vec{r},0\right)=C_{0}=1\text{mg}/\text{L}$ and the boundary condition was to maintain concentration at the boundary. (B) The time evolution of colony diameter (or the maximum microbial dispersion distance) is given from simulated results (-0.5, -1.0, and -3.0 kPa, these values were chosen to cover various hydration conditions to cover globally connected, locally connected, and fragmented habitats) and experimental results (-0.5, -1.2, and -3.6 kPa) for hydrated surfaces at three values of matric potential. Lines in different colours indicate simulated results. Filled symbols indicate the experimental results from [35].

We considered microbial population dynamics at the domain scale and their spatial distributions on the rough surface. In Fig 5, simulated values of microbial colony expansion rates are given. Fig 5A shows a typical example of microbial colony dispersion pattern on the RSPM. For the colony dispersion calculation for the domain, we made no use of local self-affine surface for the local surface porosity distribution. In order to compare simulations with the experiments that used porous ceramic surface following the mono-scale size distribution without any small or large grain, we used a random distribution of surface porosities for each patch drawn from the uniform distribution, U[0,1]. On the simulated surface domain, the nutrient concentration across the domain was given to be constant, $C\left(\vec{r},t=0\right)=C_{0}=1\text{mg}/\text{L}$. We maintained the constant concentration only at the boundary of the domain, $C\left(\vec{r},t\right){|}_{\text{boundary}}=C_{0}$. 100 microbial cells were inoculated at four patches at the centre of the domain, and their dispersion is observed over the simulation time of 60 hours. The hydration conditions during the simulation time were constant and determined by a matric potential value of *ψ*_*m*_ = −0.5kPa (static hydration condition). We also measured the time evolution of the maximum microbial dispersion distance deduced from radial colony expansion rates (as in the experiments of [35] for three different matric potential (See Fig 5B). For the comparison, we used the physical properties of an individual patch and compared the heterogenous domain, preserving the average surface porosity $\bar{\Phi }$. The comparison of simulations with experimental results were in good agreement, showing that the hydration conditions control the colony expansion rates on surfaces.

Additionally, we have developed an analytical approach for the colony expansion rate for uniformly distributed surface porosity as the most simple case. In the model, the chemotactic movement of each microorganism is described by a biased-random walk. From the tumbling probability following the growth rate field as a chemotacticfield, we earlier derived the jumping probability of an individual cell, Eq (9), that can determine the microbial dispersion rate on the domain scale. For the calculation at population level, the effective average velocity under the chemotactic field is obtained by assuming isotropic movement of a cell (again, a patch is assumed to be homogenised inside). The nutrient concentration field $C(\vec{r})$ is mapped to the growth rate field, $\mu \left(C\left(\vec{r}\right)\right)\equiv \mu \left(\vec{r}\right)$, and the gradient of the growth rate field is assumed to be a chemotactic field. This allows us to simplify the individual chemotactic locomotion when multiple nutrients are considered for the microbial growth (for the detailed derivation, see S1 Text). By assuming that the mean isotropic trajectory duration, *T*(*u*), is the same as the mean run time towards the direction $\hat{x}$, where *u* is the directional cosine of the chemotactic field direction (i.e. $u\equiv \frac{\nabla \mu \cdot \hat{x}}{\left|\nabla \mu \right|}$), the effective chemotactic velocity at population level can be calculated as follows [99, 100]:

$${\vec{v}}_{\text{eff}}\left(\psi_{m}\right)=\vec{v}\left(\psi_{m}\right)\frac{\int_{-1}^{1}uT\left(u\right)du}{\int_{-1}^{1}T\left(u\right)du}=v\left(\psi_{m}\right)\frac{I_{1}\left(\alpha |\nabla \mu |\right)}{I_{0}\left(\alpha |\nabla \mu |\right)}\frac{\nabla \mu }{\left|\nabla \mu \right|}\equiv v\left(\psi_{m}\right)\vec{R_{c}}\left(\alpha ,\mu \right),$$ (13)

where *v*(*ψ*_*m*_) is the mean swimming velocity of a cell under capillary pinning force, *T*(*u*) = *t*_0_ *e*^*α*|∇*μ*|*u*^ is the running time in direction *u* with *t*_0_, the mean run time in the absence of a chemical attractant, and *I*_*ν*_(*x*) is the modified Bessel function of the first kind. Here we introduced the chemotactic retardation factor *R*_*c*_(*α*, *μ*) that controls the effective swimming speed as a result of chemotaxis. The chemotactic factor *α* is a function of *v*(*ψ*_*m*_) and |∇*μ*| as in Eq (10), which changes over time depending on the local concentration. This means that the effective velocity of chemotaxis reaches the mean swimming velocity when *α*|∇*μ*|≫1 (strong chemotaxis), and it reaches to 0 when *α*|∇*μ*|→0 (no chemotaxis; uniform distribution of direction). From the chemotactic retardation factor, expected residence time under the chemotactic field at a patch can be calculated

$$T^{*}\left(\psi_{m}\right)=\frac{\tau \left(\psi_{m}\right)l_{p}}{v^{*}{}_{\text{eff}}\left(\psi_{m}\right)}=\frac{l_{p}}{v^{*}{}_{\text{eff}}\left(\psi_{m}\right)\xi \left(\psi_{m}\right)}=\frac{{\bar{T}}_{r}\left(\psi_{m}\right)}{R_{c}\left(\alpha^{*},\mu^{*}\right)},$$ (14)

where $v^{*}{}_{\text{eff}}\left(\psi_{m}\right)$ indicates the effective velocity at steady state (i.e. *α*|∇*μ*| is constant over time) and ${\bar{T}}_{r}\left(\psi_{m}\right)$ is the averaged minimum residence time at a patch. From the mean residence time at a patch, the microbial dispersion rate, or the expansion rate of a chemotactic ring on the rough surface can be approximately written as

$$R\left(t\right)=\int_{0}^{t}T_{r}\left(\psi_{m}\right)\vec{v}{}_{\text{eff}}\left(\psi_{m},\tau \right)\cdot \frac{\nabla \mu }{\left|\nabla \mu \right|}d\tau .$$ (15)

**Fig 6:**
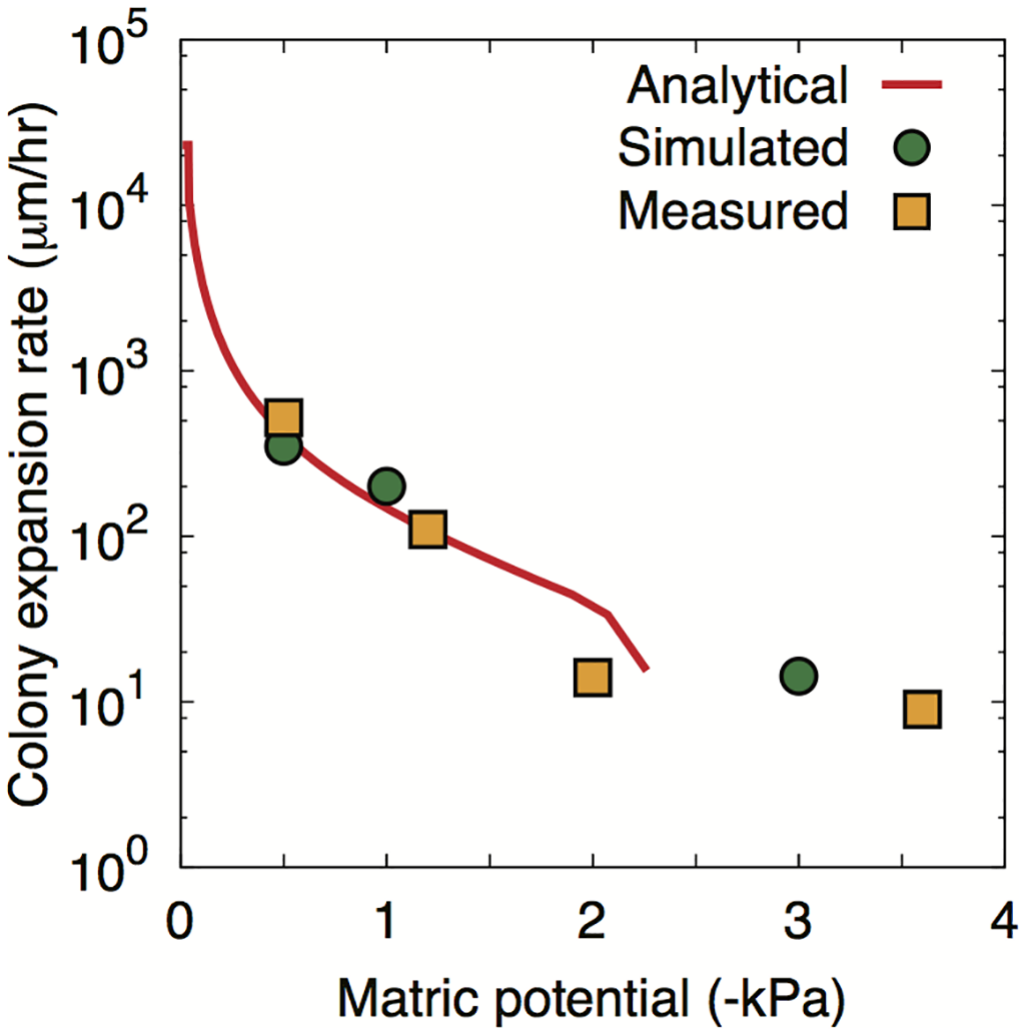
Colony expansion rate. Colony expansion rates for analytical results, surface patch model results (three matric potentials were chosen to cover various hydration conditions representing globally connected, locally connected, and fragmented habitats), and experimental results are compared. Analytic results are calculated based on Eq (15). In analytical results, the average value of the surface porosity ($\bar{\Phi }=0.5$) is used as a representative value of the domain and the expansion rate becomes zero since the flagellated movement is disabled due to the capillary forces at about −2kPa. The simulation results of RSPM show non-zero colony expansion rate up to about −3kPa because of the heterogeneity of the domain.

Fig 6 compares the analytically predicted colony dispersion rates (based on Eq (15)) with simulations and measurements by [35] as well as the simulated data from IBM. The results show that the colony expansion rate by the flagellated motility decreases exponentially from about 500*μ*m/hr at −0.5kPa to 12.5*μ*m/hr at −2kPa. Furthermore, the analytical prediction agrees with the experimental results. This implies that decomposition of physical, chemical, biological factors can be used to describe microbial dispersion. First, we calculated effective swimming velocity, which is driven from the hydrated rough surfaces with capillarity. Second, the biased random walk as chemotactic behaviour of a cell provides the net displacement towards the nutrient source. Third, considering the connectivity and tortuosity of the cell movement, it modifies the actual travelling distance that microbial cell traverses.

### Microbial community trophic interactions

An important application of the model involves cell-level trophic interactions among multiple species in the microbial community inhabiting the domain. The spontaneous spatial organisation of interdependent species has been studied for several trophic interactions based on the roughness network model [101]. This study has provided a systematic evaluation of the emergence of spatial organisation of motile microbial communities. In this work, we choose competition and mutualism as the two representative forms of microbial interactions, allowing us to elucidate the spatial organisation of different microbial consortia under the effects different surface hydrations and roughness.

**Fig 7:**
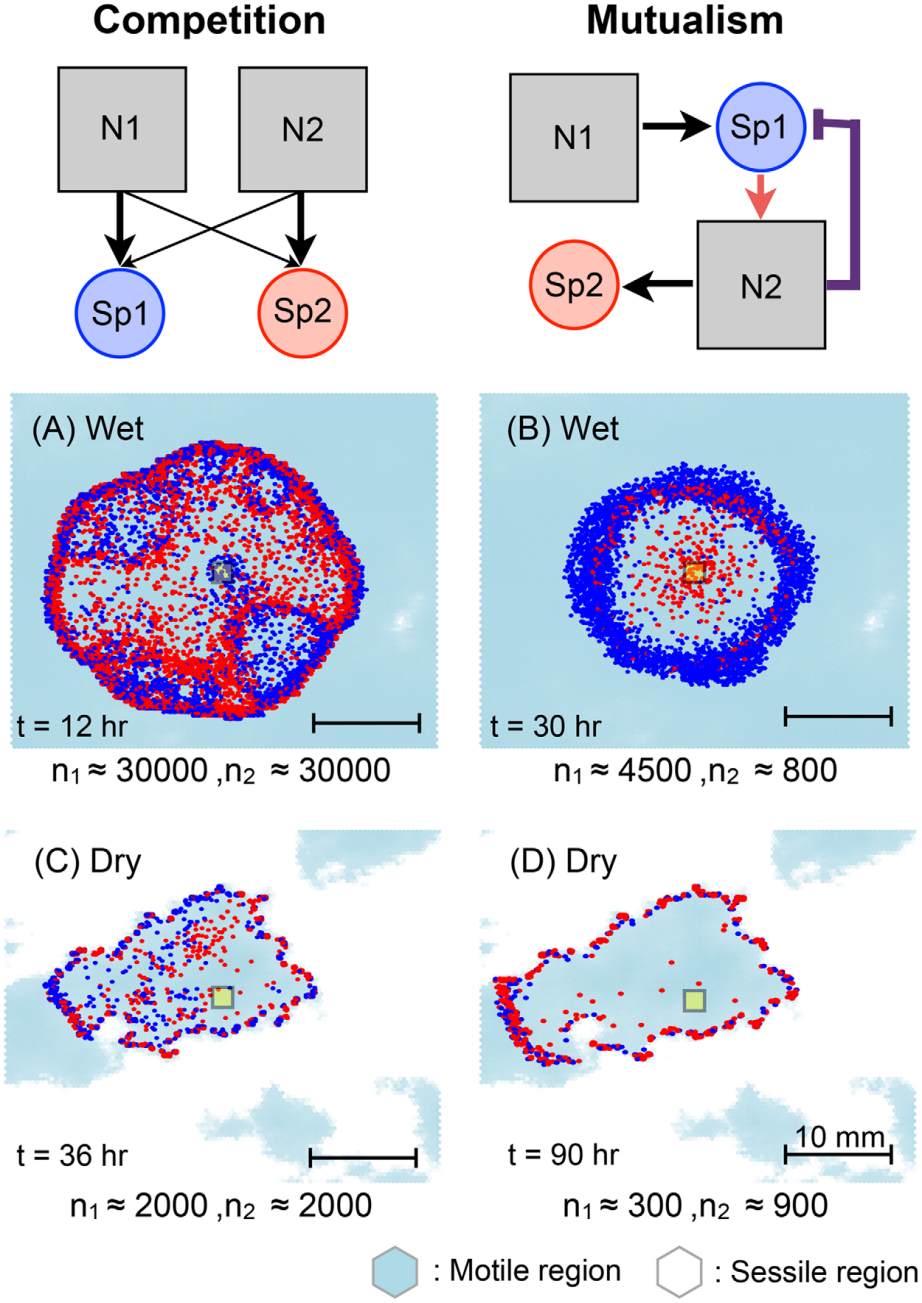
Examples of spatial patterns arising from different microbial consortia on rough surfaces for different hydration conditions. On the top figures, schematics of trophic interactions are given (competition and mutualism). Two example interactions are simulated for wet case (*ψ*_*m*_ = −0.5 kPa) and dry case (*ψ*_*m*_ = −3.6 kPa). At *t* = 0 well-mixed two populations (50 each) are inoculated at the centre (marked as a yellow square) of the roughness domain ($\bar{\Phi }=0.4$, and *D* = 1.2), Light blue indicates the distribution of aqueous habitats and blue and red dots indicate species 1 and 2, respectively. *n*_*i*_ denotes the population of species *i* in the figure at the given time. The results show that different tropic interaction give a rise to different spatial organisations. For competitive interactions, we observe segregation between two species and altering the front line on the chemotactic band (A,C) while, for mutualistic interaction, the producer (Sp1) occupies the front line of the chemotactic band and the consumer (Sp2) follows (B,D). The spatial patterns are in qualitative agreement with the previous studies on model hydrated surfaces [101].

For the competitive trophic interactions, we considered a simple case with two species and two nutrients; each species requires these two obligatory nutrients at different ratios and affinities [101]. The growth rate is determined by the limiting nutrients based on the Eq (7). Following previous studies, we assign preferences (or higher affinities) of nutrients for each species by applying different maximum yields *Y*_max,j_^*i*^ of species *i* on nutrient *j* where *i*, *j* ∈ {1, 2}. To reflect given preferences, species 1 prefers nutrient 1 and species 2 prefers nutrient 2 as shown in Fig 7 with thick arrows, maximum yields should satisfy the conditions, *Y*_max,1_^1^ < *Y*_max,2_^1^ and *Y*_max,1_^2^ > *Y*_max,2_^2^, meaning that the nutrient with lower conversion rate (maximum yield) to microbial biomass is preferred.

For the mutualistic interactions, one species consumes the by-product of the other species that, in turn, may inhibit the producer’s growth if not consumed. In other words, the initial producer (species 1) needs the other species to reduce the local concentration of its by-product and the consumer (species 2) needs species 1 for its growth. Here, we introduced the by-product yield *β* for species 1. The net growth of species 1 is given as $\mu_{\text{eff}}=\left(1-\beta \right){\tilde{\mu }}^{1}=\left(1-\beta \right)\left(\mu_{1}-m_{1}\right)$ and the by-product yield rate would be *ΔN*_2_ = *βμ*_1_ *b*_1_ *Δt*. For the sake of simplicity, we assume that other physiological parameters are equal between species and we ignore individual variances of cell size, cell velocity, and chemotactic sensitivity. Parameters used in simulations are given in S2 Table.

We generated a rough surface domain with the mean porosity $\bar{\Phi }=0.4$ and *D* = 1.2 as a representative domain and well-mixed two species populations (50 individuals per species) were inoculated at the centre (four patches) of the domain. In the simulations, the nutrient concentration across the domain was given constant, $C\left(\vec{r},t=0\right)=C_{0}=0.2\text{mg}/\text{L}$, and we maintained the constant concentration only at the boundary of the domain, $C\left(\vec{r},t\right){|}_{\text{boundary}}=C_{0}$. The hydration conditions during the simulation time were constant and determined by matric potential value of *ψ*_*m*_ = −0.5kPa and *ψ*_*m*_ = −3.6kPa for the wet and dry cases, respectively (static hydration condition). *ψ*_*m*_ = −3.6kPa is used as the drier case to guarantee the local connectivity so the spatial pattern can be observed. Fig 7 depicts the resulting spatial self-organisation for four different cases (wet-dry; competition-mutualism).

For the competitive interaction, Fig 7A and 7C, one can observe the spatial segregation of two species due to their nutrient consumption yields that lead them to occupy segregated sectors on the chemotactic band (the travelling band propagating from the inoculation point to the nutrient source that locates at the boundary of the domain). For the wet case, Fig 7A, the double travelling bands of species 1 (blue) and species 2 (red) can be observed. Travelling bands are spatially divided into several sectors, one with species 1 being ahead and species 2 following and the other with vice versa. The following species makes small sectors inside of the travelling band. Some previous studies on chemotaxis show that, under certain conditions, a second band can be seen following the first when the nutrients are not depleted by the first band and the remaining amount of nutrients are sufficient to support an internal microbial band [84, 102, 103]. These studies considered only one species and these double bands are composed of two species. In this study, we observe two-layered travelling bands of two competing species. After the first travelling band appears at the boundary, microbial cells in the band induce a steep gradient in the concentration of nutrients. Since the maximum yields of species on nutrients are different, the created gradients of each nutrients become different and it would make directionally biased motions for each species. This effect generates the second band with different species to the first band. After emergence of the two travelling bands, microbial cells within the sectors cannot experience the strong gradient of nutrients because most of the nutrients are depleted by cells in the travelling bands. Hence internally remaining cells wander within the sectors. For the drier case, Fig 7C, the double bands disappeared and a single travelling band forms with several small sectors of different species. These sectors are the results of competition between two species over the limited diffusing nutrients. As the system becomes dryer, the water film thickness is reduced affecting diffusion and thus consumption rates of nutrients. Although the gradient of nutrient concentration at the boundary of travelling band is similar to the wetter cases, the flux from the outside to the bands becomes smaller due to a thinner and fragmented water film. Each cell competes with others to occupy the front line leading to many smaller sectors at the travelling band. Since two nutrients are also obligatory for each species, these two species have to locate themselves at optimal positions that balance competition and cooperation simultaneously. The competition implies that they remain stay close to each other so that one species can consume the remaining nutrients after the consumption of other species. The results show that the differences in trophic interaction will affect to their spatial formation of colonies to optimise their consumptions based on physical processes such as diffusion of each nutrients. Unlike the results from roughness network model [101], we did not observe complete segregation since the chemotactic behaviour includes higher degree of stochasticity as microbial locomotion is described with two-dimensional biased-random walk.

For the mutualistic interaction (Fig 7B and 7D), the spatial organisation was not as strong as the competitive interactions and the pattern reflects the simplicity of the model interaction. Since species 1 (blue) degrades the primary nutrient (N1) and produces the by-product that can be used by species 2 (red) for growth, species 2 follows the chemotactic band of the species 1. In addition, the growth of species 2 requires consumption of inhibiting substance (N2), which would help species 1 by reducing local N2 concentration.

The ratio of the population size in aqueous habitats was analysed for wet and dry cases. For the wet case, Fig 7B, species 1 grows better than species 2. For the dry case, Fig 7D, the relative abundance becomes inverted. It shows that the degree of mutualistic interaction (assigned with the by-product yield *β*) and the hydration condition that mediates the effective diffusion of inhibiting substances as well as the optimal trophic distance (proposed in [101]) are closely related to the relative abundance of both species.

### Hydration effects on microbial diversity and onset of coexistence

A core question in contemporary environmental microbiology pertains to the origins and mechanisms that promote the unparalleled diversity found in soil. Using the rough surface patch model (RSPM) and the IBM (delineated above) enabled a systematic evaluation of variations in microbial community diversity with respect to hydration conditions and nutrient levels. We assigned physiological characteristics of multiple species before (numerical) inoculation on the rough surface domain. We then observed the dynamics of the species relative abundances according to the microbial diversity of the system. To avoid the complex definition of microbial species, we designed distinctive species by different Monod parameters of the growth function according to Eq (6) (Used parameters can be found in S2 Table). Due to the moderate range of growth rate values (0.44 to 1.23 hr^-1^ for *E. coli*), we used uniformly distributed values for the maximum growth rate *μ*_max_ [104]. Logarithmically distributed values for the half saturation constant *K*_*s*_ where used due to their wide spread variation (e.g. *E. coli* exhibits *K*_*s*_ values ranging from 40*μ*g.L^-1^ up to 99mg.L^-1^) [104]. Differences in assigned Monod parameters imply different nutrient consumption patterns and ecological strategies spanning the range from “pseudo-copiotrophic” to “pseudo-oligotrophic” by covering a wide range of the parameter space [105]. Other properties such as cell size and shape, motility, chemotactic sensitivity, and substrate yields were assumed equal for all species. Any functional diversity or complex trophic interactions were not included in this hypothetical scenario. As the most simplistic interaction, the model simulates the system with multiple species competing for a single nutrient diffusing through the aqueous phase (we consider other nutrients non-limiting). From the dynamics of the microbial population inhabiting the surface (species distinguished based on Eq (7)), we have tracked the time evolution of microbial diversity and the coexistence index at the steady state for various hydration conditions and two different inoculation schemes.

**Fig 8:**
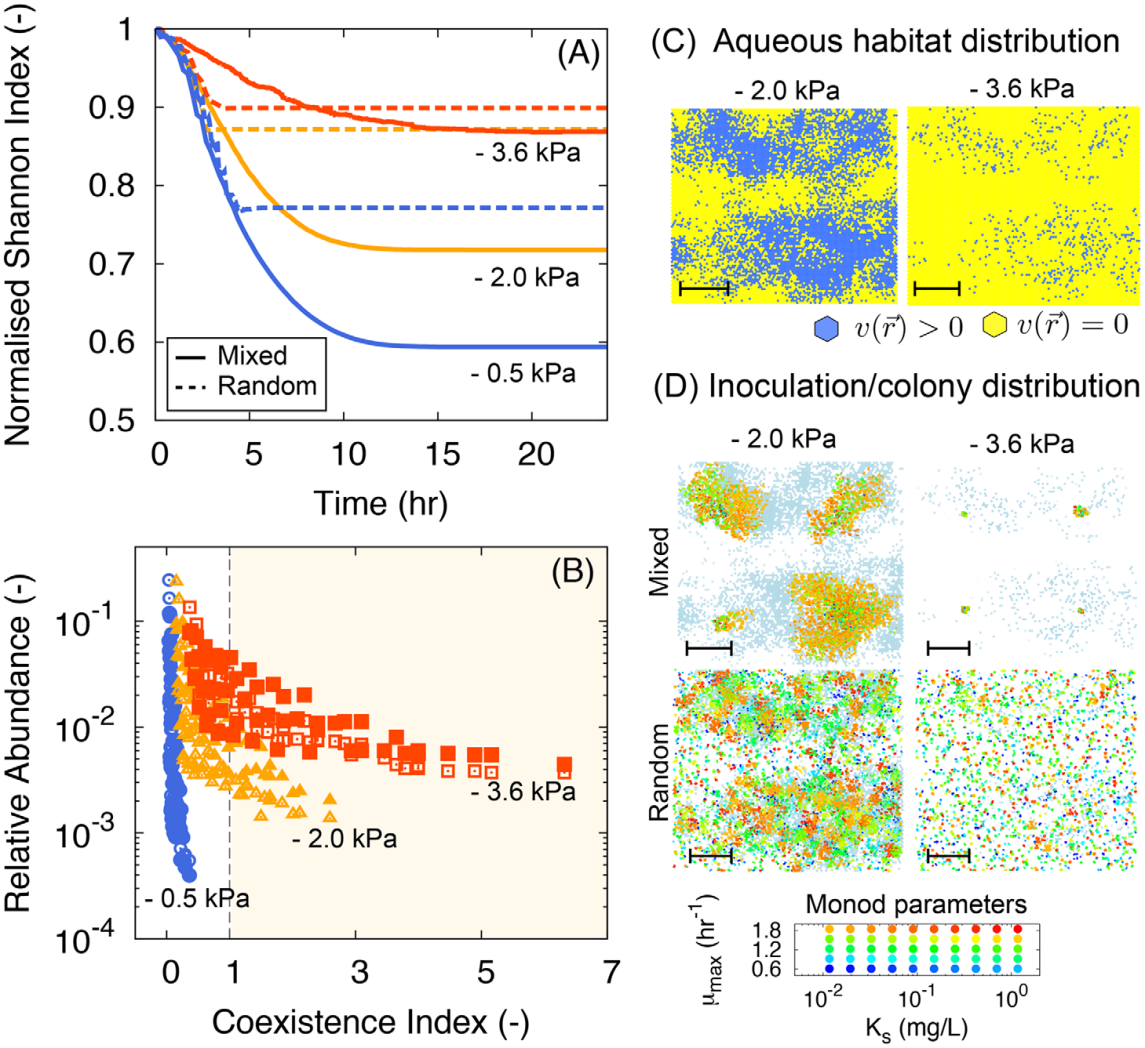
Microbial population diversity and coexistence index analysis of rough surface patch model (*D* = 1.35 and $\bar{\Phi }=0.4$, as an example case of sand) for a range of hydration conditions and associated aqueous phase connectivity. (A) Time evolution of normalised Shannon Index on the nutrient limited surface at different values of matric potential considering a population with 50 different species (differentiated by their Monod growth parameters). (B) Relative abundance rank is plotted with the coexistence index following [110] when the population sizes reach to the steady state with nutrient limited condition (at *t* = 24hr). Different line colours indicate hydration conditions of the surface, *ψ*_*m*_ = −0.5kPa in blue (wet), *ψ*_*m*_ = −2.0kPa in yellow (intermediate), and *ψ*_*m*_ = −3.6kPa in red (dry). For each hydration condition, we tested two different inoculation schemes; (1) well-mixed population inoculations (shown in solid lines and empty symbols), and (2) random inoculation for the entire domain (shown in dashed lines and filled symbols). An example of aqueous habitat distribution is given for two different matric potential in Fig 8C. Typical microbial colony distributions for four different cases (wet-dry; mixed-random) at *t* = 18hr are shown in Fig 8D. White-grey-blue scale on the background show the the microbial swimming speed field (representing aqueous habitats) and circles in various colours at each patch represent the relative population sizes and colours indicate different species with different growth patterns according to the Monod Parameters, *K*_*s*_ and *μ*_max_, shown in the graph below. The scale bars indicate 10mm. Results are obtained under the competitive interaction over a single limiting nutrient among 50 different species. As the system desiccates, the microbial diversity (Shannon Index) becomes higher. It implies that species evenness is higher when system is dry and the coexistence index becomes larger than unity (marked as yellow region in 8B). Random inoculation of microbial cells exhibits higher diversity indices suggesting that pre-colonisation of slow-growing species derives benefits from a fragmented aqueous habitat.

We employed a normalised Shannon index as an indicator of the microbial diversity on the surface:

$$H_{D}\left(t\right)=-\frac{1}{\ln N_{s}}\sum_{i=1}^{N_{s}}p_{i}\left(t\right)\ln p_{i}\left(t\right).$$ (16)

where the index is defined with the relative abundances $p_{i}\left(t\right)\equiv \frac{N_{i}\left(t\right)}{\sum_{i=1}^{N_{s}}N_{i}\left(t\right)}$, where *N*_*i*_(*t*) is the total number of individuals (living cells) of species *i* ∈ {1, ⋯, *N*_*s*_} at time *t* and *N*_*s*_ is the number of species on the domain. The Shannon index represents the diversity of the microbial community including the richness and population evenness [106–109]. In our simulations, the number of species is fixed (no extinction or migration), thus the richness is assumed to be constant. normalised Shannon index reflects the changes in evenness for different conditions (*H*_*D*_ = 1 indicates the highest evenness and all populations are equally distributed for the entire domain.). In Fig 8A, the time evolution of normalised Shannon index is given for three hydration conditions (shown in different colours) and two inoculation schemes (shown in different line types). For the “mixed” inoculation scenario, a balanced mixture of the 50 species was introduced to the surface at 4 locations, whereas for the “random” scenario, the same number of individuals were randomly distributed across the simulation domain. Results suggest that the system exhibits higher diversity/evenness when the domain is drier (*ψ*_*m*_ = −3.6kPa) as a result of the interplay among shortened dispersal range, degree of aqueous habitat fragmentation, and decrease of nutrient flux owing to thinning water film thickness. In addition, random inoculation over the entire domain shows higher diversity (dashed lines), compared to the well-mixed population inoculation (solid lines) at the same hydration condition since the roughness of the surface allows slow-growing species to colonise a certain patch in long term by sheltering them from the competition with fast-growing species. Fig 8D shows representative examples of microbial colony distribution for four different cases. When *ψ*_*m*_ = −2.0kPa (relatively wet case with locally connected aqueous habitats), one can see the strongest competitor or the fastest growing species (with the lowest *K*_*s*_ and the highest *μ*_max_; marked as orange circles) shows the highest abundance over the domain when well-mixed populations are inoculated. On the other hand, for the random inoculation, patchy distributions of various species are observed.

A biophysical index for the onset of coexistence has been proposed by [110] based on the ratio of the microbial generation length (the distance traversed by a bacterial cell along the surface during one generation—from cell division to the next) to the effective linear size of the connected aqueous cluster. This metric is expected to vary with soil hydration conditions and surface properties that affect film thickness and microbial growth rates facilitated by diffusion [101, 110]. The index links the soil hydration conditions with micro-scale aqueous habitats fragmentation. The fragmentation of habitats inhibits microbial dispersion and growth rates of microbial populations cohabiting soil surfaces, thus highly promoting microbial coexistence. Following previous studies [110], we have adopted the coexistence index (CI) for the rough surface patch model. We measured the coexistence index at a state where nutrient concentration limits microbial growth of all species (on average). In other words, for a closed system with a certain amount of nutrients (i.e., no nutrient flux from outside of the domain), the population reaches a stationary state limited by nutrients. Fig 8B depicts the relative abundance rank with the coexistence index for different hydration conditions showing clearly that the population gradually becomes more even as the surfaces dries and connected aqueous habitats become smaller and more fragmented as expected from theory [110]. The significant part of the current work is the wide coverage of the Monod parameters. For the wet case, one can observe that the coexistence index of all the species (regardless of their relative abundance) lie below the unity (blue lines for *ψ*_*m*_ = −0.5kPa). It means that the dispersal rate of all species is short compared to the range of interaction (via diffusion and uptake of the nutrient at the same/connected aqueous habitat). Even at *ψ*_*m*_ = −3.6kPa, some species still exhibits the low coexistence index while some species with high CI (larger than unity, implying coexistence).

### Surface roughness (soil texture) effects on microbial diversity

**Fig 9:**
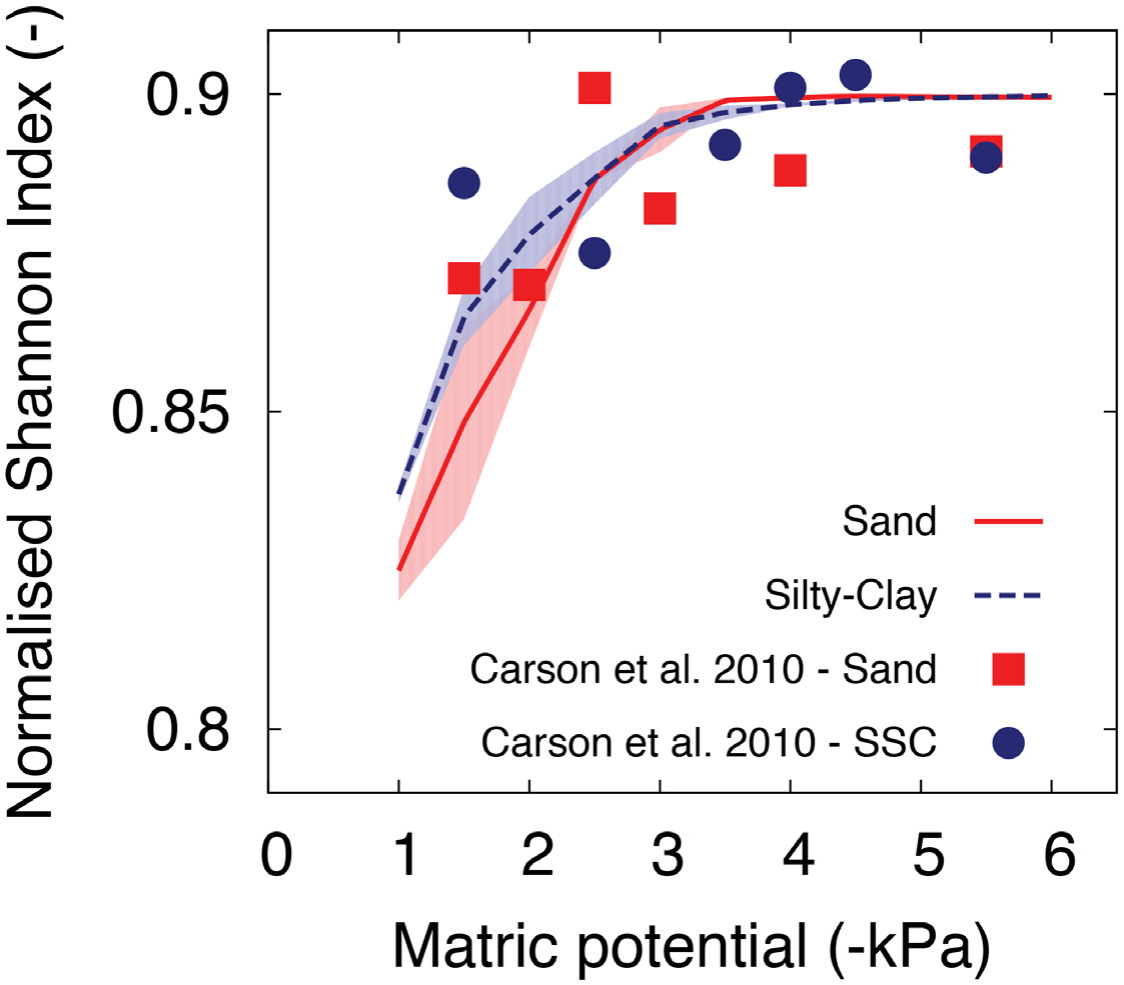
Roughness effects on microbial population diversity for different hydration conditions. Normalised Shannon index, Eq (16), calculated for two domains representing different soil textures. Different fractal dimensions are assigned for sand (*D* = 1.35) and sand+slity-clay (*D* = 1.65). Simulated results are shown in red solid line and blue dashed line for sandy surface and sand (90%)+silty-clay (10%), respectively. The values were calculated from three different rough surface domains with same roughness parameters and the shaded areas represent 1 s.t.d. Model predictions of normalised Shannon index agree with the experimental data of [115] and show a decrease in microbial population evenness as the domain becomes wet (less negative matric potential values).

In the previous section, we have shown effects of hydration conditions on microbial diversity in soils (represented as rough surface domain). The result explicitly supports the ecological theory of non-competitive diversity pattern induced by spatial isolations owing to the low connectivity [111]. Previous studies have shown that soil bacterial diversity is highly affected by particle size distribution and its relation to the fraction of fine particles in soil such as silt or clay [112, 113]. Furthermore, in terms of microbial community structure in soil, it has been shown that the similarities of the communities both within and between habitats are strongly determined by soil texture rather than vegetation type or drainage conditions [114]. The work of [115] compared the changes in microbial diversity of two soil textures (sand (100%) and sand (90%)+silt-clay (10%)) under different matric potentials. They have measured the water filled pore space (WFPS) as an indicator of the pore connectivity and have shown that bacterial diversity is strongly correlated with WFPS for two cases with different soil textures. We have compared our model and their experiments to see the effect of soil texture on microbial diversity in soils for various hydration conditions (Fig 9). For the simulated results, we have chosen different fractal dimensions to represent two different soil textures; *D* = 1.35 for sand *D* = 1.65 for 10% silty-clay [49] and the surface porosity was similar for both (i.e. $\bar{\Phi }=0.4$). We have used the same species distribution as presented in previous section; 50 species were inoculated at randomly distributed locations and the normalised Shannon index was calculated when the population sizes reach at the stationary state limited by nutrients. The results show that the model was capable of capturing the observed trends in population evenness (expressed as normalised Shannon Index) as a function of soil hydration status. The effect of surface roughness becomes important at matric potential values higher (wetter) than about *ψ*_*m*_ = −3.0kPa. Sandy surface (smooth) supports the lower evenness compared to the silty-clay (rough) surfaces. These results could be interpreted that presence of larger pores or surface regions filled with water provide advantage to more fit species with higher growth rate and lower population evenness. At the same time, rough surface with higher fraction of small obstacles (the contribution of silt-clay sized particles) would reduce the interaction degree by increasing tortuosity of the microbial dispersal pathways (see Fig 2 to compare the landscapes of the domains with different fractal dimensions), which results in higher evenness in the silty-clay domain even under wet conditions.

The experimental data were obtained from an indigenous bacterial community in field soil that undoubtedly contained a diverse array of substrates and complex trophic interactions. Although the simulated results from RSPM assumes the simplest case of competitive interaction, the model provides the comparable range of evenness degree and its changes in different hydration conditions, thus confirming previous findings (e.g., Fig 8) that as a soil becomes wetter, species with better fitness characteristics may express their physiological advantages and become dominant as a result of enabled motility, aqueous habitat connectedness, and higher nutrient transport. Conversely, when the soil dries, less interactions between species are expected (due to the disabled motility-attached to the surface), which will allow species with lower fitness to inhabit locally without competing with others with higher fitness.

## Discussion

The study presented a new and scalable modelling approach (termed the rough surface patch model, RSPM) for quantitative representation of microbial life on hydrated rough (soil) surfaces. The key element of water retention is encapsulated in patch roughness properties that collectively honours soil water retention properties and aqueous phase distribution with matric potential. Two roughness parameters, fractal dimension *D* and surface porosity Φ were used to derive the scale invariant surface pore size distribution. By using the probability density of surface pore sizes in a patch, the effective water film thickness is obtained as an indicator of roughness and regulator of nutrient diffusion and microbial dispersion for various hydration conditions. The approach provides a scalable domain (via patch size) that preserves cell-level interactions without explicit representation of pore level aqueous phase distribution that limited the scales of previous models. Despite the simplification of soil surface structure with patch representation, the model manages to capture the effects of complex aqueous phase on nutrient transport, microbial growth and community interactions. The simple representation of surfaces as patches enable simple computations for ecological modelling at sub-metric scales and for days to months time frames. Finally, larger simulation domain permits considerations of temperature and hydration gradients known to affect microbial ecology near interfaces (i.e., soil surface, desert crust etc.) [16, 116]. Patchy distribution of the microbial community and spatial heterogeneity can also be connected to the bacterial biodiversity across scales [15].

The modelling framework employs Individual based modelling (IBM) on the RSPM to describe cell-level microbial interactions in heterogeneous and time-variant environments. The IBM formulation provides an important tool to mechanistically account for multiple species growth with various biological characteristics and their trophic interactions [69]. The IBM also provides a simple means for considering individual cell motion. Each individual motion is influenced by local environments (at patch scale) and the dispersion of the population (at domain scale) emerges as an integrated effect of nutrient diffusion processes, biological growth, and chemotactic behaviour. The previous works on roughness networks succeed in showing numerically and analytically that the behaviour of population, such as the self-organisation that arises from the collection of individual movements [37, 89, 101]. This allows insight into the relation between dynamics on microscopic as well as macroscopic levels. IBM is a useful tool to bridge the gap between individual cell response and emergent behaviour of populations for various environmental conditions. However, the approach involves heavy computational burden in maintaining each individual life history as the domain and time span increase. Thus, individual based modelling at scales larger than a few centimetres remain limited. There are several attempts at dealing with large numbers of individuals such as using “super-individuals” or reducing a spatial model to a representative space [117]. Although these approaches show a promise in computational biology, the upscaling from cell level to population behaviour remains poorly understood.

Based on the analysis at the population level, the importance of patch size in the model becomes apparent. This is because the approach considers averaging (with respect to surface properties and reducing aqueous phase distribution to film thickness) across length scales varying from 10^−7^ to 10^−3^m. There are several criteria with respect to upscaling microbial growth on the patch model: First, the size of patches should be larger than a few micrometres considering the niches of microorganisms. Since the physical size of microorganisms is about 1 ∼ 10*μ*m, the size of patch should be larger than this to host several individuals. Second, considering that a patch is a homogenised subdomain, the diffusion rate of nutrients is also an important factor for choosing the size of an element. The diffusion coefficient of nutrients is of the order of 10^−10^m^2^/s. When the size of a patch is *l*_*p*_, the time for a nutrient molecule to diffuse through a patch is about $t=l_{p}^{2}/4D$ which should be comparable with the persistence time for a microbial population. Assuming that the colony expansion rate is around 5 mm/hr ≈ 10^−6^m/s (the wet case) [89], the size of patch should be around 1mm. This may define an upper limit for the patch and model scale. Although the system size is scalable for the physical properties, the length scale of the microbial dispersion limits the size of a spatial element. However, compared presently available network models mimicking a small pore size explicitly [37, 40, 41], the patchy approach enables us to upscale up to a relatively larger scale.

The new descriptions for the microbial growth and the growth rate dependent chemotaxis enable the model to be extended with multiple nutrients and multiple species. However, in IBM, assigning biological properties for multiple species is straightforward. The advantage of RSPM is the physically based description for the habitat fragmentation and connectivity that maintain salient physical features of real hydrated surfaces. Mathematical models of competitive interactions among multiple species predict that consumer species cannot coexist in excess of the number of limiting resources at equilibrium [76, 118, 119]. These models are often applied in non-spatial and homogeneous habitats for all species, in other words, the model describes essentially all-to-all interactions among individuals in well-mixed conditions. Some models show that spatial subdivision allows global coexistence of competitors [73, 75–77, 120]. In such cases that have enough nutrients to support many species, multiple species can coexist globally due to spatial fragmentation. Since the surface patch model already includes the aqueous habitat connectivity locally and globally, it allows us to investigate the effect of hydration on connectivities of such habitats, and consequently coexistence of multiples species (non-competitive diversity pattern) at larger scales.

By using the applicability of multiple species on rough surface, we have applied RSPM to examine spontaneous emergence of the spatial organisation of two species on rough surfaces. The result has shown that different trophic interactions can give rise to different spatial organisation patterns of microbial communities. The model highlights the interplay between nutrient availability through diffusion and microbial chemotactic behaviour. Especially, the spatial organisation exhibits different patterns, such as double or single travelling band, patchy distribution, and alignment along the aqueous habitats, that can be controlled by surface roughness or hydration conditions. We explored the narrow range of the hydration conditions (that enables the flagella movements; see Fig 4) since we only considered the motile microorganisms and their organisation through their chemotactic behaviour. The model can be extended by including sessile microorganisms or different types of cell motions (such as gliding, shoving) and localised nutrient sources to mimic natural environments.

A systematic evaluation of microbial diversity on rough surfaces has been explored with the RSPM-IBM model. The results elucidate the close relation between hydrated surface properties and microbial cell interactions. Although previous studies have provided the relations between the availability of water and microbial diversity [10, 121, 122], the present model provides the physical link between water content (a bulk soil variable) and water film thickness (where microbial activity and trophic interactions occur). Prior models of IBM implemented in pore-networks have provided an elegant approach to quantify the microbial interaction at micro-scale by using ordinary percolation theory on regular networks [41, 101, 110]. These studies have shown that pore-connectivity at micro-scale plays a pivotal role by regulating substrate diffusion rate, microbial dispersal length, and optimal trophic distance between different species. In this study, we extended these approaches on networks and extended it by using percolation processes on self-affine hydrated rough surface similar to those characterising soil grain surfaces. It allows us to track the connectivity at micro-scale and implement it at larger scales. In other words, the model considers cell-level interactions via effective water film thickness (that affects individual cell development and interactions with other cells in close proximity), which is obtained from the roughness of the domain and pore size distribution. The RSPM formulation enable consideration of soil texture effects (via roughness characteristics) on microbial diversity under varying hydration conditions. The model can be also applied to support experimental results of field observations by varying the boundary conditions of nutrient field, low-carbon surface soils/low-carbon saturated surface to examine different soil communities. The model is expected to show that the saturated subsurface soil communities possesses low Operational Taxonomic Unit (OTU) diversity and low evenness due to competitive interaction, spatial isolation explains diversity patterns in the low carbon surface soils [123].

Since the up-scalable model allows the examination of the dynamics of microbial life over month long time scales, survival strategies can be investigated by including dormancy or sporulation in IBM procedure [124]. Water availability during prolonged desiccation affects microbial communities, since the access to nutrients becomes more limited due to thinner water films, the nutrient flux reduces and the motility and metabolic activities cannot be supported [125]. When long-term desiccations and sporadic wetting events are considered in the simulation, survival strategies in the model can be necessary [126, 127]. Another application in the planning is to use the model to capture the dynamics of communities forming desert crusts [128–131], where vertical gradients of water, temperature and light govern population dynamics and spatial distribution.

In summary, we developed a rough surface patch model to describe microbial life in soils with a possibility of upscaling spatially to cm scales and temporally to the scale of months. The model predicts the effective water film thickness distribution at a given hydration condition over the domain, therefore microbial dispersion, carrying capacity of patch, and aqueous habitats distribution. The model possesses high applicability, quantifying spatial-organisation of multiple species on hydrated rough surfaces and measuring microbial diversity in soil under hydration cycles over months, and examining the survival strategies during prolonged desiccation. From the model predictions and comparisons with other models and experiments, we have shown the necessity of describing microbial life in soils at pore-scale and thus the importance of scalability from at pore-scale to soil representative volumes. The model serves as a bridge connecting the spatial complexity of hydrated rough soil surfaces and the motile microbial community activity based on aqueous habitat connectivity. It can be a beneficial tool to answer the questions in soil microbial ecology for the extremely high biodiversity observed at all scales, in particular, the physical composition of soil surfaces would explain the effect of abiotic factors on microbial interactions and their evolution in terms of water and nutrient availability.

## Supporting Information

S1 Table Parameters for rough surface domain. (PDF)

S2 Table Parameters for Individual Based Modelling (IBM). (PDF)

S1 Text Model description. (PDF)
