# Supplementary material for: Individual-Based Model of Microbial Life on Hydrated Rough Soil Surfaces
Source: PLoS One. 2016 Jan 25;11(1):e0147394. doi: 10.1371/journal.pone.0147394 (PMC4726620; doi:10.1371/journal.pone.0147394)
Supplement: S1 Table — (PDF) [file pone.0147394.s001.pdf]

S1 Table

Table 1. Parameters for rough surface domain

Notations	Parameters	Units	Values
r_{min}	minimum size of roughness element	m	10^{-7}
r_{max}	maximum size of roughness element	m	10^{-3},[a]
D	fractal dimension for pore size distribution	-	Vary[b]
$\bar{\Phi}$	mean surface porosity	-	Vary[b]
l_p	size of a patch	m	5×10^{-4}

Parameters used to generate roughness domain.

[a] We have used 10^{-3}m for all simulations as the largest roughness domain except for the comparison with data of [1] to consider their sample size, 50×10^{-3}m.

[b] Values of D and $\bar{\Phi}$ vary for different simulations and the values are chosen under considerations of experimental data to be compared (such as for Tuff rock (Fig. 3 in the main text), $D = 1.4$ and $\Phi = 0.1$ are used as a single patch and for sandy soil (Fig. 9 in the main text) $D = 1.35$ and $\bar{\Phi} = 0.4$ are used). Used values are given in the caption of each figure.

References

[1] Tokunaga TK, Wan J. Water film flow along fracture surfaces of porous rock. Water Resources Research. 1997;33(6):1287–1295.
