# Supplementary material for: Individual-Based Model of Microbial Life on Hydrated Rough Soil Surfaces
Source: PLoS One. 2016 Jan 25;11(1):e0147394. doi: 10.1371/journal.pone.0147394 (PMC4726620; doi:10.1371/journal.pone.0147394)
Supplement: S2 Table — (PDF) [file pone.0147394.s002.pdf]

S2 Table

Table 2. Parameters for Individual Based Modelling (IBM)

Notation	Parameter	unit	value
${\mu_{\max}}^i$	specific growth rate	hr^{-1}	Vary[a]
K_s^i	half-saturation constant	$mg.L^{-1}$	Vary[b]
$Y_{\max}$	apparent yield	$g.g^{-1}$	0.44[c]
α_m	ratio for maintenance rate	-	0.0129
$\bar{V_B}$	median cell volume	fl	0.4
$V_{\mathrm{B,d}}$	cell volume at division	fl	$2\bar{V_B}/1.433$
$V_{\mathrm{min,d}}$	minimal active cell volume	fl	$\bar{V_B}/5$
ρ	cell density (dry mass)	$fg.fl^{-1}$	290
R	size of microbial cells	μm	1
D_0	nutrient diffusion coefficient	$mm^2.hr^{-1}$	2.4
v_0	cell velocity at bulk water	$mm.hr^{-1}$	50.4
χ_0	chemotactic sensitivity	$mm^2.hr^{-1}$	180
β	by-product yield	$g.g^{-1}$	0.8

Parameters used to simulate microorganisms. Most of parameters for individual cells are chosen from [1].

[a] $\mu_{\max} = 1.23hr^{-1}$ is used for a single species and mutualistic trophic interaction. For the evaluation of microbial diversity, values are chosen uniformly spaced values in $\mathcal{U}[0.5\mu_{\max}, 1.5\mu_{\max}]$.
[b] $K_s = 1.17mg.L^{-1}$ is used for a single species. For the mutualistic trophic interaction, $K_{s,1} = K_{s,2} = K_{I,1} = 10^{-3}mg.L^{-1}$ are used. For the evaluation of microbial diversity, values are logarithmically spaced values in $\mathcal{U}[10^{-2}K_s, K_s]$.
[c] $\mu_{\max}$, K_s and $Y_{\max}$ for the Fig. 7A and Fig. 7C in the main text (Competitive trophic interaction) were used differently following the work of [2].

References

[1] Kreft JU, Booth G, Wimpenny JWT. BacSim, a simulator for individual-based modelling of bacterial colony growth. Microbiology. 1998;144(12):3275–3287.

[2] Wang G, Or D. Trophic interactions induce spatial self-organization of microbial consortia on rough surfaces. Scientific Reports. 2014;4.
