# Supplementary material for: Individual-Based Model of Microbial Life on Hydrated Rough Soil Surfaces
Source: PLoS One. 2016 Jan 25;11(1):e0147394. doi: 10.1371/journal.pone.0147394 (PMC4726620; doi:10.1371/journal.pone.0147394)
Supplement: S1 Text — (PDF) [file pone.0147394.s003.pdf]

S1 Text: Model description

Individual-Based Model of Microbial Life on Hydrated Rough Soil Surfaces

Minsu Kim[1,*], Dani Or[1]

[1] Soil and Terrestrial Environmental Physics (STEP),
Department of Environmental Systems Sciences (USYS),
ETH Zürich, 8092 Zürich, Switzerland

The rough surface patch model (RSPM) is a model for patchy hydrated surfaces defined with distributions of available water which sustains microbial life. The two-dimensional patchy domain representation helps to simplify complex soil structure and still captures not only aqueous phase configuration but also nutrient transport and microbial activities.

A patch. A patch represents a certain area on the surface and is assumed to be a uniform domain with several representative measures (i.e. homogeneous inside). Embracing the surface pore size distribution and its scale invariance property, the roughness of each patch is defined and can be rewritten as multi-scale percolation systems or pore-solid-fractal (PSF) model [1–3]. Roughness of each patch is characterised with a fractal dimension D for the pore-size distribution and the surface porosity Φ. When the size distribution of surface pore volume follows a power-law with a fractal dimension D (i.e. $N(r) \sim r^{-D}$), the probability that a point on the surface belongs to an angular pore with size X in the interval $[r, r + \delta r]$ can be written as

$$\Pr[r \leq X \leq r + \delta r] = \Phi \int_{r}^{r+\delta r} \mathcal{N}(X) dX \sim \Phi r^{-D} \delta r, \tag{1}$$

where $\mathcal{N}(r)$ is the probability density function of surface pore size r

$$\mathcal{N}(r) = \frac{1}{A} r^{-(D+1)}. \tag{2}$$

A is the normalising constant of the distribution, $A = \int_{r_{\min}}^{r_{\max}} \mathcal{N}(r) dr$. $r_{\min}$ and $r_{\max}$ are length-scale cutoffs: $r_{\min}$ is set to be 10^{-7} m to represent the minimum size of physical elements on the rough surface (related to the size of clay particle) and $r_{\max}$ is given 10^{-3} m indicating the maximum size of elements. These cutoffs are necessary for the model not only to avoid the

divergence problem but also to describe the rarity of the large-scale structure and the minimum size of roughness scale. Eq. (1) shows the surface porosity, Φ, is the proportion of angular pores on the smooth surface and the fractal dimension, D, indicates the relative effects of large-scale pore structure.

The effective water film thickness and the degree of saturation. The aim of the new model is to calculate representative measures to describe hydration status of soil rough surface, such as effective water film thickness and saturation degree. Unlike usual approaches in fractal models, this model includes corner effects of angular pores. In PSF model and Brooks-Corey model, corner effects of pores are ignored in calculations of water retention properties [2, 4–6]. In these models, each pore gets completely desaturated after the critical matric potential with an assumption that shape of pores are spheres. It can be a reasonable approach to achieve water retention property of bulk soils in terms of quantitative measures. In reality, however, pores possess angular shapes rather than spheres. Angular shapes enable soil surface to hold substantial amounts of water and it enhances the hydraulic connectivity at the low saturation [7, 8].

Let's assume that we have a small surface domain with size $\alpha {l_p}^2$, which corresponds to a patch in the model. l_p is the length scale of the patch and α is the shape factor; for square patch, $\alpha = 1$; for triangular patch, $\alpha = \frac{\sqrt{3}}{4}$. This surface might be smooth or rough. A completely smooth surface is a domain without any surface pores and roughness (i.e. $\Phi = 0$). To build a rough surface, we assume that there are two different states on the surface, angular voids (pores) and smooth surface (solids). Starting with a completely smooth surface without any voids, we build pore or solid sections in different sizes following a size distribution, $\mathcal{N}(r)$. The fractions p, s, and f (where $p + s + f = 1$) of the total area correspond to the proportion of pores, solids, and undetermined or fractal, respectively, by borrowing the concept of fractal sections in the multi-scale percolation systems [1]. Here, we keep the state, undetermined or fractal, to include sub-structures at all scales. The surface porosity can be calculated as $\Phi \equiv \frac{p}{p+s}$ in the continuum limit without the lower cutoff (i.e. $r_{\min} \to 0$). Accordingly, the number density of pores and solids of size r can be written

$$\mathcal{N}_p(r) = N_p^0 \frac{p}{f} \mathcal{N}(r) \sim \Phi \mathcal{N}(r), \quad (3)$$

$$\mathcal{N}_s(r) = N_s^0 \frac{s}{f} \mathcal{N}(r) \sim (1 - \Phi) \mathcal{N}(r), \quad (4)$$

where N_p^0 and N_s^0 are normalising constants. Here, we made an assumption that mass fractal dimension and the pore fractal dimension are the same.

We obtain the amount of water held in a single pore with the size r from two distinctive physical processes, 1) capillary water at corners obtained from the Young-Laplace equation, 2) absorbed water on the surface due to van der Waals interactions. For a simple representation of angular pore, we assume that a pore element with size r is a square pyramid with the height H and the base r. For generalising the model, H can be another variable which can be a function of r or constant in the model. The shape of pores can be also different kinds of polyhedrons, such as cubes or tetrahedrons, or can be irregular. In our model, we simply choose a square pyramid to reflect the real geometry of surface roughness and to simplify calculations. For solid elements, it is assumed to be a completely smooth surface so only absorbed water film would exist on the solid fraction. When a matric potential, ψ_m, is given as an environmental condition, the radius of meniscus curvature would be $R_\mu(\psi_m) = -\frac{\sigma}{\psi_m}$ where σ is surface tension of water. The absorbed water film thickness would be $h_\mu(\psi_m) = \left(\frac{A_{svl}}{6\pi\psi_m}\right)^{1/3}$ where A_{svl} is Hamaker constant. The matric potential determines the critical pore size, $r_c(\psi_m) = 2(R_\mu(\psi_m) + h_\mu(\psi_m))$, to get a pore desaturated. In other words, pores with size smaller than $r_c(\psi_m)$ would be saturated and pores with sizes above the critical value would be desaturated, with some capillary water remaining due to corner effects. For a pore with size r and height H, the amount of water which is held by capillarity is

$$\mathcal{V}(r,\psi_m) = \begin{cases} \mathcal{V}(r,\psi_m)^s = \frac{1}{3}Hr^2 & \text{where } r \leq r_c(\psi_m) \\ \mathcal{V}(r,\psi_m)^d = \frac{1}{3}Hr^2\Theta_r(r,\psi_m) & \text{where } r \geq r_c(\psi_m) \end{cases}, \tag{5}$$

where Θ_r is the saturation degree of an individual pore with size r,

$$\Theta_r(r,\psi_m) = \left(\frac{r_c}{r}\right)^3 + 3(4-\pi)\left(\frac{R_\mu}{r}\right)^2\left(1-\frac{r_c}{r}\right) + 6\left(\frac{h_\mu}{r}\right)\left(1-\frac{r_c^2}{r^2}\right). \tag{6}$$

The expected value of the total amount of water in the domain can be calculated following the probability distribution, Eq. (2),

$$\overline{\mathcal{V}}(\psi_m) = \int_{r_{\min}}^{r_{\max}} [p\mathcal{V}(r,\psi_m) + sh_\mu r^2]\mathcal{N}(r)dr. \tag{7}$$

Expected surface area of the patch is

$$\overline{\mathcal{A}} = \int_{r_{\min}}^{r_{\max}} (p+s)r^2\mathcal{N}(r)dr. \tag{8}$$

The effective water film thickness of each patch can be simply defined as $\overline{\mathcal{V}}(\psi_m)/\overline{\mathcal{A}}$

$$w_{\text{eff}}(\psi_m) = \frac{\overline{\mathcal{V}}(\psi_m)}{\overline{\mathcal{A}}} = \frac{\int_{r_{\min}}^{r_{\max}}[\Phi\mathcal{V}(r,\psi_m) + (1-\Phi)h_\mu r^2]r^{-(D+1)}dr}{\int_{r_{\min}}^{r_{\max}} r^2 r^{-(D+1)}dr}. \tag{9}$$

The expected saturation degree of a patch can be written

$$\Theta(\psi_m) = \frac{\int_{r_{\min}}^{r_{\max}}[\Phi\mathcal{V}(r,\psi_m) + (1-\Phi)h_\mu r^2]r^{-(D+1)}dr}{\int_{r_{\min}}^{r_{\max}}[\Phi\frac{1}{3}r^2 H(r) + (1-\Phi)h_\mu r^2]r^{-(D+1)}dr}. \tag{10}$$

Eq. (9) and Eq. (10) show that the physical measures of the effective water film thickness and the saturation degree of each patch can be calculated solely by values Φ and D. When we consider the shape factor $H(r)$ is the same as r, extra parameters are only cutoff values, $r_{\min}$ and $r_{\max}$.

In this work, we only consider the physical property of rough surface and its water-holding capacity. In the model, effects of surfactants (surface active agents) can be also included. The main property of surfactants is to lower the surface tension or to increase the contact angles [9,10]. For instance, Surfactin from *Bacillus subtilis* is known to change the surface tension of water from 72mM.m^{-1} to around 27mM.m^{-1} [9]. In Figure A, effects of lowering surface tension on effective film thickness and microbial swimming speed are given. In the figure, we fixed the contact angle as 0°.

Connectivity of within patch aqueous habitats. Notwithstanding the averaging associated with patch description, we seek to retain certain physical traits for a patch without detailed modelling of the patch roughness. Hence, the degree of aqueous habitat connectivity in a patch is described based on accessible surface pores (represented as films of certain thickness) that supports flagellated motility. The accessibility of an individual pore can be determined from the effective water film thickness of individual pore, $\overline{H}_{\text{eff}}(r,\psi_m)$. When it is larger than the size of microbial cell, $R \equiv 1\mu m$, this pore is accessible for microorganisms

$$\overline{H}_{\text{eff}}(r,\psi_m) = \frac{\mathcal{V}(r,\psi_m)}{r^2} = \frac{1}{3}H(r)\Theta_r(r,\psi_m) \geq R. \tag{11}$$

When $H(r) = r$, the critical pore size, r_a^c, for the accessibility where $\overline{H}_{\text{eff}}(r_a^c,\psi_m) = R$, can be exactly calculated from a positive root of the quadratic equation by rearranging the Eq. (11). The probability of occupation of the aqueous habitat area, a_H at ψ_m would be

$$p(\psi_m) = \frac{\overline{a}_H}{\mathcal{A}_p} = \frac{\int_{3R}^{r_a^c(\psi_m)} p\overline{H}_{\text{eff}}(r,\psi_m)r^2\mathcal{N}(r)dr}{\int_{r_{\min}}^{r_{\max}}(p+s)\overline{H}_{\text{eff}}(r,\psi_m)r^2\mathcal{N}(r)dr} = \Phi\frac{\int_{3R}^{r_a^c(\psi_m)}\mathcal{V}(r,\psi_m)\mathcal{N}(r)dr}{\int_{r_{\min}}^{r_{\max}}\mathcal{V}(r,\psi_m)\mathcal{N}(r)dr}. \tag{12}$$

Here, we used total accessible area of the patch, $\mathcal{A}_p$, as the expected area in the patch. In addition, when $r_a^c(\psi_m) < 3R$, the numerator is assumed to be zero. We determine the local connectivity $\xi(\psi_m,\vec{r})$ of the patch at $\vec{r}$ by using the occupation probability of aqueous habitats $p(\psi_m)$ and the global percolation probability $P(\psi_m)$ which is determined by the largest cluster of aqueous

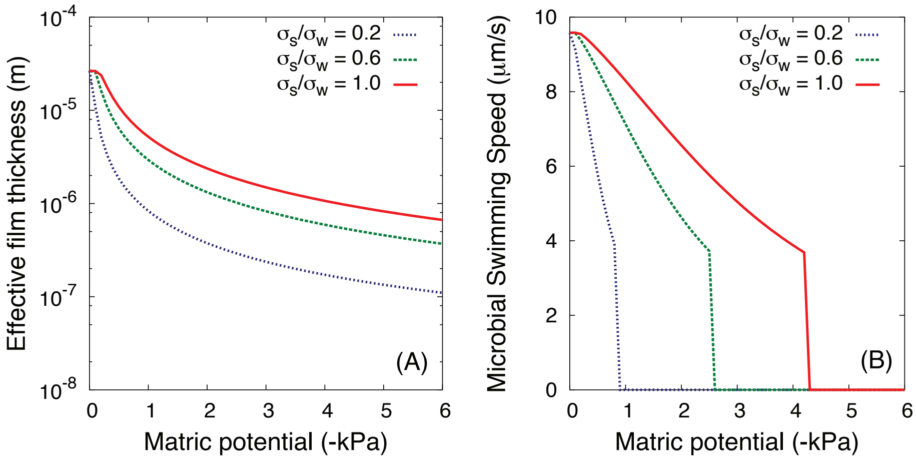

Figure A. Effects of lowering surface tension on effective water film thickness and microbial swimming speed. We have compared the effect of surface tension on effective water film thickness of the surface that affects microbial swimming velocity on a patch ($D = 1.8$ and $\Phi = 0.4$). The surface tension of water is $\sigma_w = 72$mM.m^{-1} and the changed surface tension under the effect of surfactants is σ_s. Three different cases are given, $\sigma_s = 0.2\sigma_w, 0.6\sigma_w$, and σ_w. (A) The effective water film thickness decreases when surface tension is lowered as a result of surfactant production at a given matric potential. (B) Microbial swimming speed is also slower when surfactants are produced by microorganisms.

habitats at domain scale.

$$\xi(\psi_m, \vec{r}) = \begin{cases} P(\psi_m) & \text{if } p(\psi_m, \vec{r}) > p_c(\vec{r}) \\ p(\psi_m, \vec{r})P(\psi_m) & \text{elsewhere} \end{cases},$$

where $p(\psi_m, \vec{r})$ as statistically averaged occupation probability of aqueous region in a patch and it is calculated as the expected value of the pore area where microbial motility is enabled. In the present work, we consider the percolation processes on the self-affine surface that have been analytically and numerically investigated [11–14]. Previous studies have shown that the percolation threshold p_c on self-affine surface is dependent on Hurst's exponent H (roughness parameter) and p_c is a stochastic variable with a mean value (ensemble averaged) $\langle p_c\rangle(H)$ and a variance $\sigma(H)$ regardless of system sizes. The mean value of $\langle p_c\rangle(H)$ monotonically decreases with H such that $\langle p_c\rangle(H = 0) = 0.5$ and $\langle p_c\rangle(H = 1) = 0.386$ [15]. Thus, we draw a certain local percolation threshold value $p_c(\vec{r})$ for each patch [12]. Eventually, these considerations are used to classify a patch with respect to flagellated motility as "motile" when film thickness is sufficient for motion, and the hydrated roughness is connected, otherwise a patch is declared "sessile" and not cell motion is allowed until hydration conditions change.

Diffusion process on rough surface patch model In this section, we provide the method for the numerical calculation to obtain the flux of the nutrient at each site and the time evolution of the concentration field. According to Fick's law, the flux can be obtained as

$$\vec{J}(\vec{r}, t) = -D(\vec{r})\nabla C(\vec{r}, t), \tag{13}$$

where $\vec{J}(\vec{r}, t)$ is the flux due to the gradient of nutrient concentration $C(\vec{r}, t)$. $D(\vec{r})$ is the diffusion coefficient at $\vec{r}$. Macroscopically, the value would be determined based on the distribution of roughness elements. However, in terms of nutrient transport in the model, we already include spatial effects in the effective water film thickness, thus the diffusion coefficient at bulk water D_0 is used for the calculation. The rate of the nutrient change at time t can be obtained from the divergence theorem,

$$\frac{dN(\vec{r}, t)}{dt} = \int_{\mathcal{V}} \nabla \cdot \vec{J}(\vec{r}, t) d\vec{r}, \tag{14}$$

$$= \int_{\mathcal{A}} \vec{J}(\vec{r}, t) \cdot d\vec{a}, \tag{15}$$

where $\mathcal{V}(\vec{r})$ indicates the patch at position $\vec{r}$ and $d\vec{a}$ is the surface area that the nutrient moves in/out due to Fick's law.

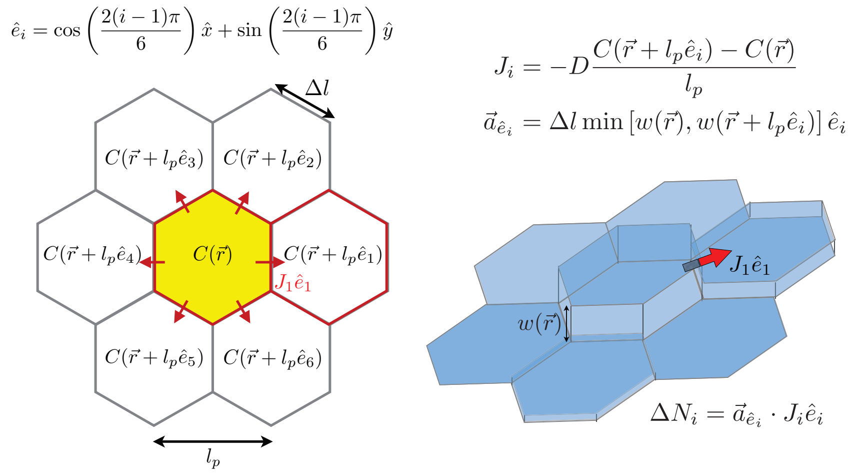

Figure B. A schematic diagram of the flux to/from patch at the position $\vec{r}$ on the hexagonal lattice.

In the numerical calculation, this integration has to be discretised. When the water film thickness at the given patch at position $\vec{r}$ is given as $w(\vec{r})$, the cross section area that the nutrient moves can be assigned as

$$\vec{a}_{\hat{n}} = \Delta l w(\vec{r})\hat{n}, \tag{16}$$

where $\hat{n}$ is the normal vector of the cross section area and Δl is a length of the edge and is related to the size of a patch. In our numerical simulation, we use the hexagonal lattice. The hexagonal lattice is better suited to describe microbial behaviour in a probabilistic model since all the neighbours are at the same distance. Figure B shows a detailed schematic diagram of the calculation for the flux of one element on the hexagonal lattice. The nearest neighbour vectors on a hexagonal lattice are denoted as $\hat{e}_i$ where $i \in [1, \cdots, 6]$. For example, the flux $\vec{J} \cdot \hat{e}_i$ denotes the flux from the current patch to the nearest neighbour $\hat{e}_i$. When the effective water film thickness is different to the neighbour, the minimum value of the film thicknesses of the adjacent pair would determine the cross sectional area,

$$\vec{a}_{\hat{e}_i} = \Delta l \min\left[w(\vec{r}), w(\vec{r}+l_p\hat{e}_i)\right]\hat{e}_i. \tag{17}$$

Equation (15) as a discretised expression can be written as

$$\frac{\Delta N(\vec{r},t)}{\Delta t} = \sum_{i=1}^{6} \vec{J} \cdot \vec{a}_{\hat{e}_i}. \tag{18}$$

This leads to the next time step,

$$N(t+\Delta t) = N(t) - \Delta N(t), \tag{19}$$

$$C(t+\Delta t) = \frac{N(t+\Delta t)}{V}, \tag{20}$$

where V is the volume of the given patch and the position vector $\vec{r}$ is rewritten with indices of the lattice.

Microbial growth (metabolism). On the soil rough surface domain, microbial activity is added by using individual based modelling (IBM) [16]. Microbial growth of an individual cell can be written as

$$\begin{aligned} \tilde{\mu}b = \frac{db}{dt} &= \left(\mu_{\max}\min[\{\frac{C_1}{K_s^1+C_1}, \frac{C_2}{K_s^2+C_2}, \cdots\}] - m\right) b, \\ \tilde{\mu} &= \mu_{\max}\min[\{\frac{C_1}{K_s^1+C_1}, \frac{C_2}{K_s^2+C_2}, \cdots\}] - m = \mu - m, \end{aligned} \tag{21}$$

where b is the biomass of the cell and its net growth rate is denoted $\tilde{\mu}$. The net growth rate is comprised with two factors, anabolism and maintenance. Anabolism could be interpreted as conversion of nutrient to cell biomass with the rate μ which is a function of nutrient concentrations, C_i, and limited with the maximum specific growth rate $\mu_{\max}$. Here, we used the min function from separate Monod growth terms to indicate the growth rate controlled by limiting nutrients. When the concentration of certain nutrient i satisfies the condition, $C_i \gg K_s^i$ for any time, this nutrient i does not restrict the growth rate of the microorganisms. Maintenance of a cell is simply given with a constant maintenance rate m.

Microbial growth rate can be determined by their substrates that can be used for growth. However, there are some substances that are toxic for cells so it inhibits their growth. For this case, the Eq. (21) can be extended as

$$\mu = \mu_{\max}\min[\{\frac{C_1}{K_s^1+C_1}, \frac{C_2}{K_s^2+C_2}, \cdots\}]\min[\{\frac{K_I^1}{K_I^1+C_1}, \frac{K_I^2}{K_I^2+C_2}, \cdots\}], \tag{22}$$

where K_I^j is the inhibition coefficient with respect to the nutrient j. When a species excretes by-product of their growth with rate β, we reduce the growth rate of the sce

Cell division. Cell division process is considered in IBM. The descriptive Danachie model is applied to estimate cell volume [17]. The cell volume of an agent is given as $V = \rho b$ with cell density ρ. When the cell volume V becomes greater than the volume at division, $V_{d,\min}$, the cell produces two identical daughter cells in juxtaposition and the biomass of each daughter cell is given as a half of the biomass of their mother cell.

Death. If an agent is under starvation condition ($\tilde{\mu} < 0$), the agent keeps shrinking and its biomass decreases due to maintenance. The agent dies if its biomass falls below a minimum value, $\rho V_{\rm min}$. On death, biomass is converted back into substrate with a conversion rate $1/Y_{\rm max}$.

Swimming speed. Bacterial flagella motility on a roughness network has been studied with regard to physical properties of pore geometries [18, 19]. In these studies, the roughness network is modelled with nodes as reservoirs of nutrients and links as water channels between reservoirs. Since the physical shape of a pore is included in the model, the movement of a microorganism and its motility could embrace mechanical aspects of soil environment to microbes. The previous model successfully obtains thresholds of motility and velocity of bacteria in the channel by applying self propulsion, cell-wall interaction, and capillary pinning force

$$v(\psi_m) = v_0 \frac{F_M - F_\lambda(\psi_m) - F_c(\psi_m)}{F_M}, \tag{23}$$

where v_0 is the velocity of a cell in bulk water and ψ_m is the matric potential that controls the effective water film thickness. F_M, F_λ, F_c are self propulsion, cell-surface interaction, and capillary pinning force, respectively. Self propulsion has the same value of viscous drag force so that the velocity of the microbes can be constant in bulk water. Cell-surface interaction and capillary force are functions of the effective water film thickness.

In the current work the rough surface is modelled by patches with macroscopic properties. All the microscopic structure of pore spaces are averaged and represented as a macroscopic variable, such as effective water film thickness and saturation degree for each patch. From the effective water film thickness, we calculate the swimming speed of microorganisms by adopting Eq. (23). The equation provides the maximum swimming speed of a cell under a certain hydration condition or roughness of the surface. When the system becomes rougher or dryer, the maximum velocity of microbes decreases.

Chemotactic Motion. For the flagellated bacteria such as *E. coli*, the motion can be described as follows. A bacterium runs, moves forward linearly, with a constant velocity for a random length of time called the "running time". Then it tumbles for a random length of time, the "tumbling time", and chooses a new direction randomly and repeats the cycle. The average running time is about 1 sec in the absence of chemotaxis at bulk water, the average tumbling time is about one tenth of the running time, about 0.1 sec, and their distributions decay exponentially [20]. Essentially, the tumbling selects a new direction, but the direction is chosen randomly because the size of bacteria is too small to detect the local gradient of attractants. However, the running length

increases when the microbial cell runs in a favourable direction. In the absence of favourable directions, the movement of microbes can be described as a random walker. On the other hand, when the preference on direction exists due to chemotaxis, a biased random walk can be considered [21]. Based on the experimental results and the theory of biased random walks, the effect of chemical attractants on individual cell paths has been studied [22–24]. The mean run time τ increases exponentially with the change in the number of receptor-attractant complexes N_b [25]

$$\tau = \tau_0 \exp\left(\sigma \frac{DN_b}{Dt}\right), \tag{24}$$

where τ_0 is the mean run time in the absence of a chemical attractant and σ is the change in the mean run time of a bacterium per rate of change of bound receptors. This implies that the number of bound receptors defines the chemotactic potential. This model is known as the "receptor" model [26]. The total derivative of the number of bound receptors is

$$\frac{DN_b}{Dt} = \frac{\partial N_b}{\partial t} + \vec{\nabla} N_b. \tag{25}$$

When we consider the case with only one attractant, a single homogeneous cell receptor density at equilibrium can be written as a Monod type of interaction,

$$N_b = \frac{N_T C}{K_d + C}, \tag{26}$$

where C is the attractant concentration, K_d is the dissociation equilibrium constant, and N_T is the total number of cell receptors for the ligand. In this model, the attractant is simply a nutrient that microorganisms consume and the kinetic equation is described as a law of mass action at equilibrium (Monod equation). This implies that the dissociation constant K_d is assumed to be the same as half concentration constant K_s. Then the spatial derivation can be rewritten as

$$\vec{\nabla} N_b = \vec{\nabla}\left(\frac{N_T C}{K_s + C}\right) = \frac{N_T K_s}{(K_s + C)^2} \vec{\nabla} C. \tag{27}$$

When we consider the trophic interaction with multiple nutrients, we have to consider the gradients of all nutrients. To simplify movement, the specific growth rate would be considered instead of concentrations of all the nutrients in the trophic interaction model. We assume that the microbial organisms would direct themselves towards the higher growth rate, as a result of chemotaxis towards necessary nutrients. Thus (27) can be further rewritten

$$\vec{\nabla} N_b = \frac{N_T K_s}{(K_s + C)^2} \vec{\nabla} C \approx \frac{N_T}{\mu_{\max}} \vec{\nabla} \mu, \tag{28}$$

where $\mu = \mu_{\max} \min \left[\frac{C_1}{K_s^1 + C_1}, \frac{C_2}{K_s^2 + C_2} \right]$. So far, we have two assumptions on chemotactic movement :

(1) The chemotactic potential is defined by the number of bound receptors.

$$\phi_c(V) = N_b = \frac{N_T C}{K_d + C}. \tag{29}$$

(2) When the chemotaxis is towards several nutrients that make growth of the cell by consumptions, the change of number of bound receptors can be interpreted with the expected specific growth rate (i.e. receptor binding model and the consumption of nutrients are the same kinetics).

$$\vec{\nabla}\phi_c(V) = \vec{\nabla}N_b = a\vec{\nabla}\mu, \tag{30}$$

where a is the proportional constant.

Employing a quasi steady-state hypothesis, one can ignore the time derivative of the number of bound receptors. As a result, when the microbe runs towards the direction $\hat{x}$, the mean run time $\tau_{\hat{x}}$ would be

$$\tau_{\hat{x}} = \tau_0 \exp\left(\frac{\sigma N_T}{\mu_{\max}} \vec{\nabla}\mu \cdot \hat{x} \right). \tag{31}$$

By combing with Alt's governing equations in the biased random walk model [21], σ can be described as

$$\sigma \equiv \frac{\chi_0}{2 N_T v}, \tag{32}$$

where v is the velocity of the microbe and χ_0 is the chemotactic sensitivity. The reciprocal of the mean run time can be assumed as a tumbling probability

$$p_{t:\hat{x}} = p_0 \exp\left(-\frac{\chi_0}{2 v \mu_{\max}} \vec{\nabla}\mu \cdot \hat{x} \right), \tag{33}$$

where p_0 is the normalisation constant. Eq. (33) describes the probability of tumbling when the microbe runs to the direction $\hat{x}$. After tumbling, the microbe runs in another direction $\hat{x}'$. The probability of the new direction is independent of the gradient of the apparent growth rate.

The model aims at up-scalability of microbial life at pore scale. Describing individual tumbling in large scale is unrealistic in terms of computational time. Thus, to simplify all the processes, we approach the distribution of microbial cells using a probability distribution with an assumption that the mean displacement after a certain time is determined based on the running time distribution towards each direction. In a hexagonal lattice as a discretised case, there are six neighbours. The unit direction to each nearest neighbour can be written as $\hat{e}_i$ where $i \in \{1, 2, \cdots, 6\}$ and $\hat{e}_7$ is

defined the origin, $\hat{e}_7 \equiv (0, 0)$. The probability to move towards the direction $\hat{e}_i$, $p_i(t)$, can be determined as

$$p_i(t) = \frac{w_i e^{(\alpha \vec{\nabla}\mu(t)\cdot\hat{e}_i)}}{\sum_{j=1}^{7} w_j e^{\alpha\vec{\nabla}\mu(t)\cdot\hat{e}_j}}, \tag{34}$$

where α is the factor for the chemotactic motion,

$$\alpha \equiv \frac{\chi_0}{2\mu_{\max} v(\psi_m)}. \tag{35}$$

In the expression, $v(\psi_m)$ is the velocity of microbe at the given matric potential ψ_m. In Eq. (34), the weight factor w_i is determined by effective water film thickness $d_i(\psi_m)$

$$w_i = \frac{d_i(\psi_m)}{\sum_{j=1}^{7} d_j(\psi_m)}, \tag{36}$$

where d_7 is the water film thickness of the current patch where the cell is. We assume that the flux of the nutrients from each neighbour will weigh the chemotactic movements. When the water film thicknesses of some neighbours are thinner than the size of microbial cell, $w < R$, we modify the weight factor as follows since the patch is physically non accessible for the cell

$$w_i' = \begin{cases} \frac{d_i(\psi_m)}{\sum_{j=1}^{7} d_j(\psi_m)} & \text{if } d_i \geq R \\ 0 & \text{if } d_i < R \\ \frac{\left(\sum_{k, d_k < R} d_k(\psi_m)\right) + d_7}{\sum_{j=1}^{7} d_j(\psi_m)} & \text{if } i = 7 \end{cases}.$$

By applying this weight factor, one can make the staying probability unity when the cell is trapped in the patch due to physical barriers. On the other hand, when the structure is homogeneous, the weight factor would be cancelled out and the distribution follows only the chemotactic probability. In addition, when the chemotactic sensitivity is very low, (i.e. $\alpha \to 0$), the system becomes a pure diffusion system without chemotaxis and the microbial cells move based on the distribution of water.

Based on the probability towards each direction, the expected displacement can be calculated

$$\langle \vec{X}(t) \rangle = \sum_{i=1}^{6} v(\psi)\Delta t \; p_i(t)\hat{e}_i. \tag{37}$$

Here, we do not use a continuous integration since the population size of patch and the migration rate between patch are our interest in the model. The expected displacement would be accumulated until it exceeds the patch size l_p with the tortuosity effect:

$$\left| \sum_{k=1}^{n} \vec{X}(k\Delta t) \right| > \tau(\psi_m) l_p, \tag{38}$$

where $\tau(\psi_m)$ is the tortuosity of the patch defined from roughness and connectivity. This criterion is simply given as a step function of the staying probability and it forces the agent to move to the neighbour patch if the displacement exceeds the patch length. This part can be changed to a stochastic process by applying the probability to escape from the current patch, given as $p(t) = e^{-\langle X(t)\rangle/l_p}$. The probability to move to neighbouring patch i from the current patch is determined as

$$\mathcal{T}_{\cdot\rightarrow i} = \frac{{X_{\mathrm{tot}}}^i}{\sum_{j=1}^{6} {X_{\mathrm{tot}}}^j}, \tag{39}$$

where ${X_{\mathrm{tot}}}^i = \sum_{k=1}^{n} |\vec{X}_i(k\Delta t)|$. As a result, we divide the time to stay at a given patch based on Eq. (38) and the population distribution to the neighbour patch based on Eq. (39). A diagram for the numerical calculation is given in Figure C.

References

[1] Neimark A. Multiscale percolation systems. Soviet Physics - Journal of Experimental and Theoretical Physics. 1989;96(4):1386–1396.

[2] Perrier E, Bird N, Rieu M. Generalizing the fractal model of soil structure: The pore–solid fractal approach. Geoderma. 1999;88(3):137–164.

[3] Perrier E, Bird N, Pachepsky Y, Radcliffe D, Selim H. The PSF model of soil structure: a multiscale approach. Scaling Methods in Soil Physics. 2003;p. 1–18.

[4] Bird N, Perrier E, Rieu M. The water retention function for a model of soil structure with pore and solid fractal distributions. European Journal of Soil Science. 2000;51(1):55–63.

[5] Kewen L, et al. Theoretical development of the Brooks-Corey capillary pressure model from fractal modeling of porous media. Society of Petroleum Engineers. 2004;.

[6] Ghanbarian-Alavijeh B, Millán H, Huang G. A review of fractal, prefractal and pore-solid-fractal models for parameterizing the soil water retention curve. Canadian Journal of Soil Science. 2011;91(1):1–14.

[7] Ransohoff T, Radke C. Laminar flow of a wetting liquid along the corners of a predominantly gas-occupied noncircular pore. Journal of Colloid and Interface Science. 1988;121(2):392–401.

[8] Or D, Tuller M, et al. Flow in unsaturated fractured porous media: Hydraulic conductivity of rough surfaces. Water Resources Research. 2000;36(5):1165–1177.

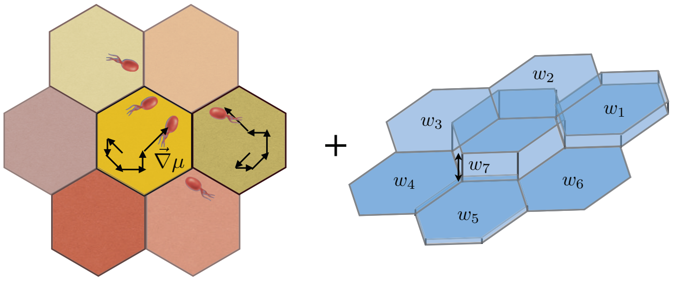

$$p_i(t) = \frac{w_i e^{(\alpha \vec{\nabla}\mu(t)\cdot\hat{e}_i)}}{\sum_{j=1}^{7} w_j e^{\alpha\vec{\nabla}\mu(t)\cdot\hat{e}_j}} \qquad \alpha \equiv \frac{\chi_0}{2\mu_{\max} v(\psi_m)}$$

		Expected displacement
t	$\vec{X}(t) = \vec{v}(\psi_m)\Delta t$	$\langle\vec{X}(t)\rangle = \sum_{i=1}^{6} p_i(t)X_i(t)\hat{e}_i$
$\vdots$		
$t = t_0 + n\Delta t$	$X^i_{\rm tot} = \sum_{k=1}^{n} \lvert X_i(k\Delta t)\rvert$	$\left\lvert\sum_{k=1}^{n}\vec{X}(k\Delta t)\right\rvert > \tau(\psi_m)l_p$
$t = t_0 + \tau_s$	Probability to jump to the neighbour patch i $\mathcal{T}_{\cdot\to i} = \frac{{X_{\rm tot}}^i}{\sum_{j=1}^{6}{X_{\rm tot}}^j}$	

Figure C. A diagram for the numerical calculation of individual movement. The biased probability of movement is given as a function of the effective water film thickness and the chemotactic factor α. The displacement during Δt towards each direction is given as $|v(\psi_m)|\Delta t$. When the cumulative expected displacement exceeds the distance between patches, $\tau(\psi_m)l_p$, the microbes will move to the other patch based on the transition probability $\mathcal{T}_{\cdot\to i}$.

[9] Christofi N, Ivshina I. Microbial surfactants and their use in field studies of soil remediation. Journal of Applied Microbiology. 2002;93(6):915–929.

[10] Rockhold ML, Yarwood R, Niemet MR, Bottomley PJ, Selker JS. Considerations for modeling bacterial-induced changes in hydraulic properties of variably saturated porous media. Advances in Water Resources. 2002;25(5):477–495.

[11] Isichenko MB. Percolation, statistical topography, and transport in random media. Reviews of Modern Physics. 1992;64(4):961.

[12] Schmittbuhl J, Vilotte JP, Roux S. Percolation through self-affine surfaces. Journal of Physics A: Mathematical and General. 1993;26(22):6115.

[13] Du C, Satik C, Yortsos Y. Percolation in a fractional Brownian motion lattice. AIChE Journal. 1996;42(8):2392–2395.

[14] Sahimi M. Non-linear and non-local transport processes in heterogeneous media: from long-range correlated percolation to fracture and materials breakdown. Physics Reports. 1998;306(4):213–395.

[15] Prakash S, Havlin S, Schwartz M, Stanley HE. Structural and dynamical properties of long-range correlated percolation. Physical Review A. 1992;46(4):R1724.

[16] Kreft JU, Booth G, Wimpenny JWT. BacSim, a simulator for individual-based modelling of bacterial colony growth. Microbiology. 1998;144(12):3275–3287.

[17] Donachie W, Robinson A. Cell division: parameter values and the process. In: Neidhardt C, Ingraham J, Brooks Low K, Magasanik B, Schaechter M, Umbarger H, editors. *Escherichia coli* and *Salmonella typhimurium*: Cellular and Molecular Biology. vol. 2. 1st ed. Washington, DC: ASM Press; 1987. p. 1578–1593.

[18] Wang G, Or D. Aqueous films limit bacterial cell motility and colony expansion on partially saturated rough surfaces. Environmental Microbiology. 2010;12(5):1363–1373.

[19] Long T, Or D. Microbial growth on partially saturated rough surfaces: Simulations in idealized roughness networks. Water Resources Research. 2007;43(2):W02409.

[20] Berg H. Bacterial microprocessing. In: Cold Spring Harbor symposia on quantitative biology. vol. 55. Cold Spring Harbor Laboratory Press; 1990. p. 539–545.

[21] Alt W. Biased random walk models for chemotaxis and related diffusion approximations. Journal of Mathematical Biology. 1980;9(2):147–177.

[22] Ford RM, Lauffenburger DA. Measurement of bacterial random motility and chemotaxis coefficients: II. Application of single-cell-based mathematical model. Biotechnology and Bioengineering. 1991;37(7):661–672.

[23] Rivero MA, Tranquillo RT, Buettner HM, Lauffenburger DA. Transport models for chemotactic cell populations based on individual cell behavior. Chemical Engineering Science. 1989;44(12):2881–2897.

[24] Lovely PS, Dahlquist F. Statistical measures of bacterial motility and chemotaxis. Journal of Theoretical Biology. 1975;50(2):477–496.

[25] Berg HC, Brown DA. Chemotaxis in Escherichia coli analysed by three-dimensional tracking. Nature. 1972;239(5374):500–504.

[26] Hillen T, Painter KJ. A user's guide to PDE models for chemotaxis. Journal of Mathematical Biology. 2009;58(1-2):183–217.
